# Proceedings of the 13th annual conference of INEBRIA

**DOI:** 10.1186/s13722-016-0062-9

**Published:** 2016-09-19

**Authors:** Rod Watson, James Morris, John Isitt, Pablo Barrio, Lluisa Ortega, Antoni Gual, Kenneth Conner, Tracy Stecker, Stephen Maisto, Sophie Paroz, Caroline Graap, Véronique S Grazioli, Jean-Bernard Daeppen, Susan E Collins, Nicolas Bertholet, Jennifer McNeely, Vlad Kushnir, John A. Cunningham, Iain K Crombie, Kathryn B Cunningham, Linda Irvine, Brian Williams, Falko F Sniehotta, John Norrie, Ambrose Melson, Claire Jones, Andrew Briggs, Peter Rice, Marcus Achison, Andrew McKenzie, Elena Dimova, Peter W Slane, Véronique S. Grazioli, Susan E. Collins, Sophie Paroz, Caroline Graap, Jean-Bernard Daeppen, Stéphanie Baggio, Marc Dupuis, Joseph Studer, Gerhard Gmel, Molly Magill, Véronique S. Grazioli, Robert J. Tait, Lucinda Teoh, Erin Kelty, Elizabeth Geelhoed, David Mountain, Gary K. Hulse, Elina Renko, Shannon G. Mitchell, David Lounsbury, Zhi Li, Robert P. Schwartz, Jan Gryczynski, Arethusa S. Kirk, Marla Oros, Colleen Hosler, Kristi Dusek, Barry S. Brown, Deborah S. Finnell, Aisha Holloway, Li-Tzy Wu, Geetha Subramaniam, Gaurav Sharma, Sara Wallhed Finn, Sven Andreasson, Robert D. Dvorak, Matthew P. Kramer, Brittany L. Stevenson, Emily M. Sargent, Tess M. Kilwein, Sion K. Harris, Lon Sherritt, Sarah Copelas, John R. Knight, The Partnership for Adolescent Substance Use Research, Noreen D Mdege, Jim McCambridge, Gallus Bischof, Anja Bischof, Jennis Freyer-Adam, Hans-Juergen Rumpf, Niamh Fitzgerald, Lisa Schölin, Paul Toner, Jan R. Böhnke, Laura J. Veach, Olivia Currin, Leigh Z. Dongre, Preston R. Miller, Elizabeth White, Emily C. Williams, Gwen T. Lapham, Jennifer J. Bobb, Anna D. Rubinsky, Sheryl L. Catz, Susan Shortreed, Kara M. Bensley, Katharine A. Bradley, Joanna Milward, Paolo Deluca, Zarnie Khadjesari, Rod Watson, Stephanie Fincham-Campbell, Colin Drummond, Kathryn Angus, Linda Bauld, Sophie Baumann, Katja Haberecht, Inga Schnuerer, Christian Meyer, Hans-Jürgen Rumpf, Ulrich John, Beate Gaertner, Marion Barrault-Couchouron, Marion Béracochéa, Vincent Allafort, Valérie Barthélémy, Hervé Bonnefoi, Emmanuel Bussières, Véronique Garguil, Marc Auriacombe, Marianne Saint-Jacques, Michel Dorval, Katia M’Bailara, Lidia Segura-Garcia, Nuria Ibañez-Martinez, Juan Manuel Mendive-Arbeloa, Manel Anoro-Perminger, Pako Diaz-Gallego, Mª Angeles Piñar-Mateos, Joan Colom-Farran, Marianthi Deligianni, Bertrand Yersin, Angeline Adam, Constance Weisner, Felicia Chi, Wendy Lu, Stacy Sterling, Kevin L. Kraemer, Kathleen A. McGinnis, David A. Fiellin, Melissa Skanderson, Adam J. Gordon, Jonathan Robbins, Susan Zickmund, P. Todd Korthuis, E. Jennifer Edelman, Nathan B. Hansen, Christopher J. Cutter, James Dziura, Lynn E. Fiellin, Patrick G. O’Connor, Stephen A. Maisto, Roger Bedimo, Cynthia Gilbert, Vincent C. Marconi, David Rimland, Maria Rodriguez-Barradas, Michael Simberkoff, Amy C. Justice, Kendall J. Bryant, Anne H Berman, Gillian W Shorter, Jeremy W Bray, Carolina Barbosa, Magnus Johansson, Reid Hester, William Campbell, Maria Lucia O. Souza Formigoni, André Luzi Monezi Andrade, Laisa Marcorela Andreoli Sartes, Christopher Sundström, Niels Eék, Martin Kraepelien, Viktor Kaldo, Claudia Fahlke, Lynn Hernandez, Sara J. Becker, Richard N. Jones, Hannah R. Graves, Anthony Spirito, Silke Diestelkamp, Lutz Wartberg, Nicolas Arnaud, Rainer Thomasius, Jacques Gaume, Véronique Grazioli, Cristiana Fortini, Zelra Malan, Bob Mash, Katherine Everett-Murphy, Véronique S. Grazioli, Joseph Studer, M. Mohler-Kuo, Nicolas Bertholet, Gerhard Gmel, Lawrence Doi, Helen Cheyne, Ruth Jepson, Vanesa Luna, Leticia Echeverria, Silvia Morales, Teresa Barroso, Ângela Abreu, Cosma Aguiar, Duncan Stewart, Angela Abreu, Riany M. Brites, Rafael Jomar, Gerson Marinho, Pedro Parreira, J. Paul Seale, J. Aaron Johnson, Dena Henry, Sharon Chalmers, Freida Payne, Linda Tuck, Akula Morris, Cátia Gonçalves, Bettina Besser, Cristina Casajuana, Hugo López-Pelayo, María Mercedes Balcells, Lídia Teixidó, Laia Miquel, Joan Colom, Kimberly A. Hepner, Katherine. J. Hoggatt, Andy Bogart, Susan. M. Paddock, Sarah L Hardoon, Irene Petersen, Fiona L Hamilton, Irwin Nazareth, Ian R. White, Louise Marston, Paul Wallace, Christine Godfrey, Elizabeth Murray, Hana Sovinová, Ladislav Csémy

**Affiliations:** 1 Health Innovation Network, London, SE1 9BB UK; 2 Alcohol Academy, London, SM5 2PS UK; 3 Resonant, London, W1D 6HW UK; 4 GRAC, Addictions Unit, Department of Psychiatry, Clinical Institute of Neuroscience, Hospital Clínic, Barcelona, 08036 Spain; 5 Fundació Clínic Recerca Biomèdica (FCRB), RETICS (Red de Trastornos adictivos), University of Barcelona, Barcelona, 08036 Spain; 6 Department of Emergency Medicine, University of Rochester Medical Center, Rochester, NY 14642 USA; 7 Department of Nursing, Medical University of South Carolina, Charleston, SC 29425 USA; 8 Department of Psychology, Syracuse University, Syracuse, NY 13244 USA; 9 Alcohol Treatment Center, Lausanne University Hospital, Lausanne, 1011 Switzerland; 10 University of Washington-Harborview Medical Center, Seattle, WA 98195 USA; 11 Department of Population Health, New York University School of Medicine, New York, NY 10016 USA; 12 Center for Addiction and Mental Health, Toronto, M6J 1H4 Canada; 13 National Institute for Mental Health Research, Australian National University, Canberra, Australia; 14 Division of Population Health Sciences, University of Dundee, Dundee, DD2 4BF UK; 15 Nursing, Midwifery and Allied Health Professions, University of Stirling, Stirling, FK9 4LA UK; 16 Institute of Health and Society, Medical Faculty, Newcastle University, Newcastle upon Tyne, Tyne and Wear NE1 7RU UK; 17 Centre for Healthcare Randomised Trials (CHaRT), University of Aberdeen, Aberdeen, Aberdeenshire AB25 2ZD UK; 18 Institute of Health and Wellbeing, University of Glasgow, Glasgow, G12 0XH UK; 19 Division of Neuro Science, University of Dundee, Dundee, DD1 9SY UK; 20 Erskine Practice, Arthurstone Medical Centre, Dundee, DD4 6QY UK; 21 Institute of Health and Society, Newcastle University, Newcastle upon Tyne, NE1 7RU UK; 22 Department of Community Medicine and Health, Lausanne University Hospital, Lausanne, 1011 Switzerland; 23 Department of Psychiatry and Behavioral Sciences, University of Washington, Seattle, WA 98195 USA; 24 University of Lausanne, Lausanne, 1015 Switzerland; 25 Lausanne University Hospital, Lausanne, 1011 Switzerland; 26 Center for Alcohol and Addiction Studies, Brown University, Providence, RD USA; 27 Alcohol Treatment Center, Lausanne, Lausanne University Hospital, Lausanne, 1011 Switzerland; 29 National Drug Research Institute, Faculty Health Science, Curtin University, Perth, Western Australia 6102 Australia; 30 School of Psychiatry and Clinical Neurosciences, The University of Western Australia, Perth, Western Australia 6009 Australia; 31 School of Population Health, The University of Western Australia, Perth, Western Australia 6009 Australia; 32 School of Primary, Aboriginal and Rural Health Care, The University of Western Australia, Perth, Western Australia 6009 Australia; 33 Department of Emergency Medicine, Sir Charles Gairdner Hospital, Perth, Western Australia 6009 Australia; 34 Department of Social Sciences, University of Helsinki, Helsinki, 00170 Finland; 35 Friends Research Institute, Baltimore, MD 21201 USA; 36 Albert Einstein College of Medicine, Yeshiva University, Bronx, NY 10461 USA; 37 College of Global Public Health, New York University, New York, NY 10003 USA; 38 United Health Care, Baltimore, MD 21045 USA; 39 The Mosaic Group, Baltimore, MD 21210 USA; 40 Institute of Health and Society, Newcastle University, Newcastle upon Tyne, Tyne and Wear NE1 7RU UK; 41 School of Nursing, Johns Hopkins University, Baltimore, MD 21218 USA; 42 Nursing Studies, School of Health in Social Science, The University of Edinburgh, Edinburgh, EH8 9AG UK; 44 Department of Psychiatry and Behavioral Sciences, Department of Medicine and Duke Clinical Research Institute, Duke University Medical Center, Durham, NC 27710 USA; 45 Center for the Clinical Trials Network, National Institute on Drug Abuse, National Institutes of Health, Bethesda, MD 20892 USA; 46 The EMMES Corporation, Rockville, MD 20850 USA; 47 Friends Research Institute, Inc., Baltimore, MD 21201 USA; 48 Department of Public Health Sciences, Karolinska Institutet, Stockholm, 171 77 Sweden; 49 Department of Psychology, University of Central Florida, Orlando, Florida 32816 USA; 50 Department of Psychology, University of North Dakota, Grand Forks, North Dakota 58202 USA; 51 Department of Pediatrics, Harvard Medical School, Boston, MA 02115 USA; 52 Center for Adolescent Substance Abuse Research, Boston Children’s Hospital, Boston, MA 02115 USA; 53 Department of Health Sciences, University of York, York, YO10 5DD UK; 54 Department of Psychiatry, University of Luebeck, Luebeck, 23538 Germany; 55 Institute of Epidemiology and Social Medicine, University of Greifswald, Greifswald, 17489 Germany; 56 Institute for Social Marketing, UK Centre for Tobacco and Alcohol Studies, Faculty of Health Sciences and Sport, University of Stirling, Stirling, FK9 4LA UK; 58 Hull York Medical School, University of York, York, Yorkshire YO10 5DD UK; 59 General Surgery, Wake Forest School of Medicine, Winston-Salem, NC 27517 USA; 60 Group Health Research Institute, Seattle, WA 98101 USA; 61 VA HSRD Center of Innovation for Veteran-Centered and Value-Driven Research, Seattle, WA 98101 USA; 62 University of Washington, Seattle, WA 98195 USA; 63 VA San Francisco, San Francisco, CA 94121 USA; 64 University of California at Davis, Sacramento, CA 95616 USA; 65 Addictions Department, Institute of Psychiatry, London, SE58BB UK; 66 Institute for Social Marketing, UK Centre for Tobacco and Alcohol Studies, School of Health Sciences, University of Stirling, Stirling, FK9 4LA UK; 67 Institute of Social Medicine and Prevention, University Medicine Greifswald, Greifswald, 17489 Germany; 68 German Center for Cardiovascular Research, Site Greifswald, Greifswald, 17489 Germany; 69 Department of Psychiatry and Psychotherapy, Medical University of Luebeck, Luebeck, 23562 Germany; 70 Department of Epidemiology and Health Monitoring, Robert Koch Institute Berlin, Berlin, 13302 Germany; 71 Humanities and Social Sciences Group, Regional Cancer Center Institut Bergonié, Bordeaux, 33076 France; 72 Laboratory of Psychology, University of Bordeaux, Bordeaux, 33076 France; 73 Oncological Surgery and Oncology Departments, Regional Cancer Center Institut Bergonié, Bordeaux, 33076 France; 74 Department of Addiction Treatment, Charles Perrens Hospital Center, Bordeaux, 33000 France; 75 Studies and research programs in addiction diseases, Sherbrooke University, Sherbrooke, Québec J1K 2R1 Canada; 76 Department of Social and Preventive Medicine, Laval University, Québec, G1V 0A6 Canada; 77 German Centre for Cardiovascular Research, Site Greifswald, 17489 Germany; 78 Catalonia Department of Health, Public Health Agency, Government of Catalonia, Barcelona, Barcelona 08005 Spain; 79 La Mina Primary Health Center, Catalan Institute of Health, Government of Catalonia, Sant Adria de Besos, Barcelona 08930 Spain; 80 Besos Primary Health Center, Catalan Institute of Health, Government of Catalonia, Barcelona, Barcelona 08019 Spain; 81 Larrard Primary Health Center, PAMEN and Barcelona City Council, Government of Catalonia, Barcelona, Barcelona 08024 Spain; 82 Figueres Primary Health Center, Catalan Institute of Health, Government of Catalonia, Figueres, Gerona 17600 Spain; 83 Alcohol Treatment Center, Lausanne University Hospital, Lausanne, Vaud 1011 Switzerland; 84 Emergency Department, Lausanne University Hospital, Lausanne, Vaud 1011 Switzerland; 85 Department of Psychiatry, University of California, San Francisco, CA 94143 USA; 86 Division of Research, Kaiser Permanente Northern California, Oakland, CA 94612 USA; 87 Department of Medicine, University of Pittsburgh, Pittsburgh, PA 15213 USA; 88 Department of Medicine, Yale University, New Haven, CT 06520 USA; 90 Department of Medicine, Oregon Health Sciences University, Portland, OR 97239 USA; 91 Yale University School of Medicine, New Haven, CT 06510 USA; 92 Center for Interdisciplinary Research on AIDS, Yale University School of Public Health, New Haven, CT 06510 USA; 94 Yale Center for Analytic Sciences, Yale University School of Public Health, New Haven, CT 06511 USA; 95 Syracuse University, Syracuse, NY 13244 USA; 96 Veterans Affairs North Texas Health Care System and UT Southwestern, Dallas, Texas 75216 USA; 97 D.C. VAMC and George Washington University, Washington, DC 20422 USA; 98 Atlanta VAMC and Emory University School of Medicine, Atlanta, Georgia 30033 USA; 99 Michael E. DeBakey VAMC and Baylor College of Medicine, Houston, Texas 75216 USA; 100 VA NY Harbor Healthcare System and New York University, New York, NY 10010 USA; 101 VA Connecticut Healthcare System, Veterans Aging Cohort Study, West Haven, CT 06516 USA; 102 National Institute on Alcohol Abuse and Alcoholism HIV/AIDS Program, Bethesda, MD 20892-7003 USA; 103 Karolinska Institutet, Department of Clinical Neuroscience, Center for Psychiatry Research, 113 64 Stockholm, Sweden; 104 Trinity College Dublin, University of Dublin, Dublin 2, Ireland; 105 School of Health in Social Science, The University of Edinburgh, Edinburgh, EH8 9AG Scotland, UK; 106 The University of North Carolina at Greensboro, Greensboro, NC 27412 USA; 107 RTI International, Chicago, IL 60606 USA; 108 Department of Medicine, Karolinska Institutet, Stockholm, 17177 Sweden; 109 Research Division, Checkup & Choices, LLC, Albuquerque, NM 87112 USA; 110 Departamento de Psicobiologia, Universidade Federal de São Paulo, São Paulo, 04021-001 Brazil; 111 Departamento de Psicologia, Universidade Federal de Juiz de Fora, Juiz de Fora, 36036-900 Brazil; 112 Department of Clinical Neuroscience, Karolinska Institutet, 171 77, Solna Sweden; 113 Department of Psychology, Gothenburg University, 405 30 Gothenburg, Sweden; 114 Center for Alcohol and Addiction Studies, Brown University, Providence, RI 02912 USA; 115 Department of Psychiatry and Human Behavior, Brown University, Providence, RI 02912 USA; 116 German Center for Addiction Research in Childhood and Adolescence, University Clinic Hamburg Eppendorf, Hamburg, 20246 Germany; 117 Alcohol Treatment Center, Department of Community Health and Medicine, Lausanne University Hospital, Lausanne, 1011 Switzerland; 118 Division of Family Medicine and Primary Care, Stellenbosch University, Stellenbosch, 8000 South Africa; 119 Chronic Diseases Initiative in Africa (CDIA), Faculty of Health Sciences, Cape Town University, Cape Town, 7701 South Africa; 120 Institute of Social and Preventive Medicine, and Epidemiology, Biostatistics and Preventive Institute, University of Zurich, Zurich, 8001 Switzerland; 121 Scottish Collaboration for Public Health Research and Policy, Usher Institute of Population Health Science and Informatics, University of Edinburgh, Edinburgh, EH8 9AG UK; 122 Nursing, Midwifery and Allied Health Professionals Research Unit, University of Stirling, Stirling, FK9 4LA UK; 123 School of Psychology, National University of Mexico, Mexico City, 04510 Mexico; 124 Mental Health Nursing Department, Nursing School Coimbra, Coimbra, 3046-851 Portugal; 125 Public Health Nursing Department, Federal University of Rio de Janeiro, Rio de Janeiro, RJ 21941-901 Brazil; 126 Emergency Department, Central Hospital Dr. Ayres Meneses, São Tomé, São Tomé and Príncipe; 127 University of York, York, Yorkshire YO10 5DD UK; 128 Public Health Nursing, Federal University of Rio de Janeiro, Rio de Janeiro, RJ 21941-901 Brazil; 129 Coimbra Nursing School, Coimbra, 3046-851 Portugal; 130 Family Medicine, Mercer University, Macon, GA 31206 USA; 131 Institute of Public and Preventive Health, Augusta University, Augusta, GA 30912 USA; 132 School of Nursing, University of North Georgia, Dahlonega, GA 30597 USA; 133 College of Nursing, Mercer University, Atlanta, GA 30341 USA; 134 Department of Nursing, Armstrong Atlantic State University, Savannah, GA 31419 USA; 135 Mental Health Nursing Department, Nursing School of Coimbra, Coimbra, 3046-851 Portugal; 136 ARS-Centro, Coimbra, 3020 Portugal; 137 Department of Psychiatry and Psychotherapy, University of Luebeck, Luebeck, 23538 Germany; 138 Grup de Recerca en Adiccions Clínic (GRAC), Addictions Unit, Department of Psychiatry, Clinical Institute of Neuroscience, Hospital Clínic, Universitat de Barcelona, Red de Trastornos Adictivos (RTA), Barcelona, 08036 Spain; 139 Institut d’Investigacions Biomèdiques August Pi i Sunyer (IDIBAPS), Barcelona, 08036 Spain; 140 Fundació Clínic per la Recerca Biomèdica, Barcelona, 08036 Spain; 141 Agència de Salut Pública de Catalunya, Departament de Drogodependències, Generalitat de Catalunya, Barcelona, 08002 Spain; 142 RAND Corporation, Santa Monica, CA 90407 USA; 143 VA HSR&D Center for the Study of Healthcare Innovation, Implementation and Policy, VA Greater Los Angeles Healthcare System, Sepulveda, CA 91343 USA; 144 Department of Primary Care and Population Health, University College London, London, WC1E 6BT UK; 145 Addictions Department, King’s College London, London, WC2R 2LS UK; 146 MRC Biostatistics Unit, Cambridge, Cambridgeshire CB2 0SR UK; 148 Center for Coordination, Monitoring and Research on Alcohol and Tobacco, National Institute of Public Health, Prague, Bohemia Czech Republic 100 42; 149 Center for Epidemiological and Clinical Research of Addictions, National Institute of Mental Health, Klecany, Bohemia Czech Republic 250 67

## A1 Service evaluation of alcohol identification and brief advice (IBA) direct to the public in a novel setting

### Rod Watson^1^, James Morris^2^, John Isitt^3^

#### ^1^Health Innovation Network, London, SE1 9BB, UK; ^2^Alcohol Academy, London, SM5 2PS, UK; ^3^Resonant, London, W1D 6HW, UK

**Correspondence:** Rod Watson - rodwatson@nhs.net

*Addiction Science & Clinical Practice* 2016, **11(Suppl 1)**: A1

**Background**: Many young people in England do not use services associated with delivery of alcohol IBA (also called screening and brief intervention). The project tested whether IBA can be delivered to 18–30 year-old, on busy city streets, by trained workers who were not healthcare professionals, without framing it as an ‘alcohol reduction’ intervention. This approach may be referred to as ‘IBA Direct’.

**Materials and methods**: Numbers of participants in the intervention were recorded on a monitoring sheet, along with the individual’s gender, age and AUDIT score. The evaluator asked some participants to complete a brief, anonymous feedback form about their experience of the intervention.

**Results**: The project was delivered over 3 days, amassing a total of 24 h across 2 Saturdays and 1 Sunday in August 2015. Four workers were present on all days. In total, 402 brief interventions were completed; however, data from 379 participants were recorded. Forty-one percent were female (21 % missing data) and 42 % were aged in their teens or twenties. A participant feedback form was completed by 61 people. Ninety-three percent (n = 57) rated the service as ‘Excellent’ or ‘Good’. All respondents who answered the question on the suitability of the setting of the service (n = 58) said it was suitable. Nine out of ten respondents (n = 55) stated they would participate in this service in a public setting again.

**Conclusions**: The evaluation of this project has demonstrated the feasibility and high acceptability of IBA Direct being delivered by non-health workers to the public on the streets of London. There were high levels of engagement at each location and among those aged 18–30. Important facilitators were considered to be the ‘branding’ of the intervention and materials, for example, framed as a ‘health quiz’ not ‘alcohol reduction’ and incentives to draw people in such as free ‘mocktails’ (soft drinks).

## A2 Innovative support for the patient with alcohol dependence (SIDEAL): Pilot study of a mobile app for alcohol dependence

### Pablo Barrio^1^, Lluisa Ortega^2^, Antoni Gual^1^

#### ^1^GRAC, Addictions Unit, Department of Psychiatry, Clinical Institute of Neuroscience, Hospital Clínic, Barcelona, Barcelona, 08036, Spain; ^2^Fundació Clínic Recerca Biomèdica (FCRB), RETICS (Red de Trastornos adictivos), University of Barcelona, Barcelona, 08036, Spain

**Correspondence:** Pablo Barrio - pbarrio@clinic.cat

*Addiction Science & Clinical Practice* 2016, **11(Suppl 1)**: A2

**Background**: Information and Communication Technologies (ICT) have opened new possibilities in the field of alcohol dependence (AD). In particular, mobile applications (APPs) could provide some relevant improvements in the management of AD, as has been shown in previous studies. The aim of this study is to report the results of a pilot study testing the usability and satisfaction of a mobile APP (called SIDEAL) in AD patients.

**Materials and methods**: Adult, outpatient subjects with AD were included. SIDEAL was installed on patients’ own phones. The Time Line Follow-Back (TLFB) for the preceding 6 weeks was administered both at baseline and after 6 weeks (study end). Self-reports from the app were also assessed at study end and compared to data provided by the TLFB. An online questionnaire about usability and satisfaction was surveyed to participants after study completion. Exploratory efficacy analyses were conducted. Patients kept their usual treatment.

**Results**: 29 patients were included (mean age 48 (SD 11.3), women 50 %) Of these, 2 never used the APP and 3 only used it in the initial visit, and were excluded from analysis. Most patients (22/24) chose a consumption reduction aim, with 11 patients receiving nalmefene. Patients used the self-register module of the APP on average 80 % of days. The most valued modules were the consumption and medication self-register ones, as well as the weekly feedback provided by the APP about weekly rate of usage. Satisfaction was high. Significant reductions were observed in alcohol consumption (binge drinking days in the last 6 weeks declined from 25 (SD 18.6) to 5.8 (SD 8), p < 0.001, mean daily alcohol consumption in standard units declined from 6.5 (SD 4.3) to 1.9 (SD 1.8), p < 0.001). Most of days (88 %) patients achieved their self-imposed objectives.

**Conclusions**: SIDEAL is a well-accepted, highly used APP by AD patients that could improve the efficacy of AD management. Further larger, randomized studies will have to confirm these preliminary, encouraging results.

## A3 Preliminary test of a brief intervention in promoting treatment initiation in middle-aged and older adults with markedly elevated AUDIT scores

### Kenneth Conner^1^, Tracy Stecker^2^, Stephen Maisto^3^

#### ^1^Department of Emergency Medicine, University of Rochester Medical Center, Rochester, NY, 14642, USA; ^2^Department of Nursing, Medical University of South Carolina, Charleston, SC, 29425, USA; ^3^Department of Psychology, Syracuse University, Syracuse, NY, 13244, USA

**Correspondence:** Kenneth Conner - kenneth_conner@urmc.rochester.edu

*Addiction Science & Clinical Practice* 2016, **11(Suppl 1)**: A3

**Background**: There is a lack of evidence that SBIRT interventions promote treatment initiation (Glass et al. 2015), yet for some populations a focus on obtaining treatment seems essential, including middle-aged and older adults with markedly elevated AUDIT scores for whom harmful alcohol use may not be expected to remit or “mature out” with normative developmental changes.

**Materials and methods**: This is a secondary analysis of data from an RCT (Stecker et al. 2012) that examines the subsample ages 50 and older. For the RCT, participants were recruited through community advertisements. Those who had no history of alcohol treatment and scored ≥16 on the AUDIT, indicative of a need for “counseling and monitoring” (Babor et al. 2001), were eligible and assigned to a CBT treatment session or a non-treatment control. The CBT intervention lasted 45–60 min and was delivered by phone, with a focus on re-examining specific beliefs that may have served to prevent the individual from previously initiating treatment. Of the original sample (N = 196), n = 55 (28 %) were ages 50 and older, and their data were analyzed.

**Results**: Individuals in the subsample were 67 % female, 91 % white non-Hispanic, and with mean AUDIT score 24.3. At 3-month follow-up, an unadjusted logistic regression analysis showed a trend for individuals in the treatment condition to be more likely to initiate treatment, odds ratio (95 % confidence interval) = 3.85 (0.93, 16.01), p = 0.068.

**Conclusions**: These preliminary results suggest that a brief CBT intervention is efficacious in promoting treatment engagement in middle-aged and older adults that likely have significant alcohol-related problems. Future directions include assessment of AUD symptoms with a diagnostic instrument, examining whether the intervention improves drinking outcomes, and if treatment initiation serves as a mechanism for such improvement.

## A4 Qualitative evaluation of a brief harm-reduction intervention among socially marginalized substance users attending a drop-in center

### Sophie Paroz^1^, Caroline Graap^1^, Véronique S. Grazioli^1^, Jean-Bernard Daeppen^1^, Susan E. Collins^2^

#### ^1^Alcohol Treatment Center, Lausanne University Hospital, Lausanne, 1011, Switzerland; ^2^University of Washington-Harborview Medical Center, Seattle, WA 98195, USA

**Correspondence:** Sophie Paroz - sophie.paroz@chuv.ch

*Addiction Science & Clinical Practice* 2016, **11(Suppl 1)**: A4

**Background**: Although socially marginalized individuals experience severe substance-related harm, they are underserved by traditional treatments and need alternative interventions tailored to their needs. The Harm-Reduction Treatment-Brief Intervention (HaRT-BI) was developed and administrated within a Swiss drop-in center for substance users. Participants were attendees who participated in a larger study (N = 101, 16.0 % female; mean age = 38.4). At baseline and 1-month assessments, harm-reduction goals and safer-drinking strategies were elicited. The achievement of goals and use of strategies were assessed 1 and 6 months later. This qualitative study aimed to evaluate users’ experience of this intervention.

**Materials and methods:** Face-to-face semi-structured qualitative interviews were conducted with 72 participants 1 month after the end of the intervention. Participants’ experience of the HaRT-BI as well as their memory, use and experienced benefits of elicited goals and strategies were discussed. Interviews were audio recorded and transcribed. A qualitative content analysis was performed.

**Results:** Most participants reported that they appreciated the discussion of harm-reduction goals and safer-drinking strategies. They usually remembered the elicited goals and strategies over time and mentioned their supportive role in daily life. Commonly reported benefits included having the opportunity to discuss, assess and consider one’s own current substance use and quality of life. Observing one’s own achievements was a source of pride, motivation and self-confidence, whereas projecting into future realizations or coping with no achievement were experienced by some participants as emotionally difficult. Some participants reported they continued using the strategies, some adapted them to their needs and used alternative strategies aiming to reduce harm related to other substances.

**Conclusions:** The general experience of the HaRT-BI was positive. The findings shed light on participants’ desire to discuss their substance use and social situations, their ability to mobilize resources as well as to introduce changes. Findings support the need for brief harm-reduction interventions tailored for socially marginalized substance users.

## A5 **Smartphone application for unhealthy alcohol use**: **a pilot study**

### Nicolas Bertholet^1^, Jean-Bernard Daeppen^1^, Jennifer McNeely^2^, Vlad Kushnir^3^, John A. Cunningham^3,4^

#### ^1^Alcohol Treatment Center, Lausanne University Hospital, Lausanne, 1011, Switzerland; ^2^Department of Population Health, New York University School of Medicine, New York, NY 10016, USA; ^3^Center for Addiction and Mental Health, Toronto, M6J 1H4, Canada; ^4^National Institute for Mental Health Research, Australian National University, Canberra, Australia

**Correspondence:** Nicolas Bertholet - Nicolas.Bertholet@chuv.ch

*Addiction Science & Clinical Practice* 2016, **11(Suppl 1)**: A5

**Background**: Smartphone, an item people carry with them almost all the time, offers opportunities in delivering interventions for unhealthy alcohol use at the user’s convenience. We developed a smartphone application with 5 modules: 1. Personal feedback, 2. Self-monitoring of drinking, 3. Designated driver tool, 4. Blood alcohol content calculator, 5. Information. We assessed its acceptability and association between use and drinking.

**Materials and methods**: 130 adults with unhealthy alcohol use (>14 drinks/week or ≥1 episode/month with 6 or more drinks), recruited in Switzerland (n = 70) and Canada (n = 60), were offered to use the application. Follow-up was at 3 months. We assessed appreciation, usefulness and frequency of use of the modules, and drinking outcomes (drinks/week, binge). Associations between application use and drinking at follow-up were evaluated with negative binomial and logistic regression models, adjusted for baseline values.

**Results**: 48 % of participants were women, mean (SD) age: 32.8(10.0). Follow-up rate: 86.2 %. There were changes from baseline (BL) to follow-up (FU) in number of drinks/week, BL: 15.0(16.5); FU: 10.9(10.5), p = 0.0097, and binge drinking, BL: 95.4, p < 0.0001. All modules were favorably rated by those who used them: median ratings were between 6 and 8 (scale of 1–10). Except for the personal feedback module, absence of use was reported by 46.4 of participants (23 % did not use the application at all). Participants using the application more than once reported significantly fewer drinks/week at follow up (IRR = 0.69[0.51; 0.94]) but not less binge drinking (OR = 0.76[0.33; 1.74]).

**Conclusions**: A smartphone application for unhealthy alcohol use appears acceptable. Nevertheless, without prompting, its use is infrequent. Those who used the application more than once reported less drinking at follow-up. Efficacy of the application should be tested in a randomized trial with strategies to increase frequency of its use.

## A6 **Reducing alcohol consumption in obese men**: **a priority for action**

### Iain K. Crombie^1^, Kathryn B. Cunningham^1^, Linda Irvine^1^, Brian Williams^2^, Falko F. Sniehotta^3^, John Norrie^4^, Ambrose Melson^5^, Claire Jones^1^, Andrew Briggs^5^, Peter Rice^6^, Marcus Achison^1^, Andrew McKenzie^1^, Elena Dimova^1^, Peter W. Slane^7^

#### ^1^Division of Population Health Sciences, University of Dundee, Dundee, DD2 4BF, UK; ^2^Nursing, Midwifery & Allied Health Professions, University of Stirling, Stirling, FK9 4LA, UK; ^3^Institute of Health and Society, Medical Faculty, Newcastle University, Newcastle upon Tyne, Tyne and Wear, NE1 7RU, UK; ^4^Centre for Healthcare Randomised Trials (CHaRT), University of Aberdeen, Aberdeen, Aberdeenshire, AB25 2ZD, UK; ^5^Institute of Health and Wellbeing, University of Glasgow, Glasgow, G12 0XH, UK; ^6^Division of Neuro Science, University of Dundee, Dundee, DD1 9SY, UK; ^7^Erskine Practice, Arthurstone Medical Centre, Dundee, DD4 6QY, UK

**Correspondence:** Iain K Crombie - i.k.crombie@dundee.ac.uk

*Addiction Science & Clinical Practice* 2016, **11(Suppl 1)**: A6

**Background**: Obese men who drink heavily are at greatly increased risk of liver disease. This makes them a priority for intervention. Obese men may be reluctant to engage in an intervention to reduce alcohol consumption. This feasibility study developed and tested methods to overcome this challenge.

**Materials and methods**: Two recruitment strategies were used: through GP registers and by community outreach. The intervention was systematically developed based on formative research, public involvement and behavior change theory (the Health Action Process Approach). The intervention was organized in two phases: a face to face session delivered by trained lay people (Study Coordinators), followed by a series of text messages.

**Results:** In total 69 men were recruited, exceeding the intended total of 60. Both recruitment strategies were successful, but recruiting through GP registers was much less labor intensive than by community outreach. Almost all the men (95 %) were at a 19 fold increase in the risk of dying from liver disease. The men engaged enthusiastically with the intervention and most (71 %) made plans to reduce their alcohol consumption. A very high follow-up rate was achieved (98 %). The two outcome measures for a full trial were successfully measured.

**Conclusions**: The recruitment methods of this study identified men at very high risk of liver disease. There is an urgent need to intervene. This study has developed an acceptable intervention and effective methods for the conduct of a full trial.

*Trial registration*: ISRCTN55309164

## A7 **Telling a story to change behavior**: **evaluation of a narrative based intervention**

### Linda Irvine^1^, Brian Williams^2^, Falko F. Sniehotta^3^, Ambrose Melson^4^, Iain K. Crombie^1^

#### ^1^Division of Population Health Sciences, University of Dundee, Dundee, DD2 4BF, UK; ^2^Nursing, Midwifery & Allied Health Professions, University of Stirling, Stirling, FK9 4LA, UK; ^3^Institute of Health and Society, Newcastle University, Newcastle upon Tyne, NE1 7RU, UK; ^4^Institute of Health and Wellbeing, University of Glasgow, Glasgow, G12 0XH, UK

**Correspondence:** Linda Irvine - m.a.j.irvine@dundee.ac.uk

*Addiction Science & Clinical Practice* 2016, **11(Suppl 1)**: A7

**Background**: Narrative transportation, a mechanism by which an individual engages with a storyline, may assist in behavior change interventions by influencing beliefs, attitudes and intentions. This study evaluates the use of a narrative in a novel text message intervention designed to reduce binge drinking among disadvantaged men.

**Materials and methods**: The intervention was based on the Health Action Process Approach. A narrative featuring a fictional protagonist, Dave, described his journey from regular binge drinking to moderate drinking. It also included Dave’s friends who demonstrated varying degrees of success in reducing drinking. A framework, which incorporated the causal chain to behavior change, guided the construction of the narrative. The narrative was rendered into texts messages that were delivered to participants over 3 months.

**Results**: The intervention comprised 112 text messages. Characters from the narrative featured in 50 messages. Dave and his friends modeled steps to behavior change, e.g. goal setting and identifying benefits of reduced drinking. This encouraged participants to report their own experiences. They demonstrated narrative transportation by responding to the text messages. Empathy with the characters was frequently elicited: e.g. Dave’s friend’s misfortune prompted the response ‘So sorry about the news Dougie’. When Dave modeled one method to reduce alcohol consumption, one man replied ‘Tonight I drank shandy with my meal, you’re a good influence Dave!;-)’.

**Conclusions**: A high level of engagement was achieved with a narrative delivered by text message. Participants may be more receptive to health messages when modeled by characters with whom they can identify and empathize.

*Trial registration*: ISRCTN07695192

## A8 **Socially marginalized alcohol and other drug users attending a drop-in center allowing alcohol consumption and receiving a harm-reduction brief intervention onsite**: **six-month substance use outcomes**

### Véronique S. Grazioli^1^, Susan E. Collins^2^, Sophie Paroz^1^, C. Graap^1^, Jean-Bernard Daeppen^1^

#### ^1^Department of Community Medicine and Health, Lausanne University Hospital, Lausanne, 1011, Switzerland; ^2^Department of Psychiatry and Behavioral Sciences, University of Washington, Seattle, WA, 98195, USA

**Correspondence:** Véronique S. Grazioli - Veronique.Grazioli@chuv.ch

*Addiction Science & Clinical Practice* 2016, **11(Suppl 1)**: A8

**Background**: Despite their experience of substance-related harm, few socially marginalized alcohol and other drug (AOD) users access substance use treatment. Identifying alternative approaches addressing substance-related harm in this population is therefore important. This program evaluation documented substance use and quality-of-life (QoL) outcomes following exposure to such an alternative: A drop-in center allowing alcohol consumption and providing harm-reduction counseling onsite.

**Materials and methods**: Participants (N = 85) were socially marginalized AOD users (e.g., alcohol, heroin) attending a harm-reduction drop-in center in the French-speaking part of Switzerland. The Harm Reduction Treatment–Brief Intervention—(HaRT-BI), designed to elicit self-generated harm-reduction goals and discuss safer-drinking strategies, was administrated at baseline and at 1-month follow-up. Participants were administered assessments of substance use and quality of life at the baseline and 1- and 6-month follow-ups.

**Results**: A within-subjects analysis of the program was conducted. Population-averaged generalized estimating equation indicated that the passage of time in the evaluation, but not exposure to the drop-in center (i.e., months of attendance), was a significant predictor of typical (IRR = .95, SE = .02, p = .006) and peak (IRR = .96, SE = .02, p = .007) alcohol quantities, and of alcohol-related problems (IRR = .93, SE = .02, p = .002). These results indicated that, for each month of the evaluation, participants’ alcohol use and related problems decreased by 4–5 and 7 %, respectively. Drop-in center attendance predicted additional decreases in drug-related problem severity (B = −.01, SE = .01, p = .017) and improvements in mental health-related QoL (B = .95, SE = .39, p = .014).

**Conclusions**: These initial findings indicate that harm-reduction drop-in centers allowing alcohol consumption and providing onsite harm-reduction counseling represent promising interventions for socially marginalized AOD users.

## A9 **Screening for alcohol use disorder**: **The problem of subthreshold problem drinkers in DSM-5**

### Stéphanie Baggio^1^, Marc Dupuis^1^, Joseph Studer^2^, Gerhard Gmel^2^

#### ^1^University of Lausanne, Lausanne, 1015, Switzerland; ^2^Lausanne University Hospital, Lausanne, 1011, Switzerland

**Correspondence:** Stéphanie Baggio - stephanie.baggio@unil.ch

*Addiction Science & Clinical Practice* 2016, **11(Suppl 1)**: A9

**Background**: Screening for alcohol abuse is an important preventive healthcare focus for early prevention and public health planning. Alcohol use disorder (AUD) is widely used, but a non-negligible proportion of people are not symptom-free without meeting a formal diagnose of AUD. In DSM-IV classification, these undiagnosed alcohol users constituted an important numeric group more likely to develop further AUD. DMS-5 AUD was designed to provide a better coverage of subthreshold problem drinkers, but few studies investigated this topic. This study provided a screening of subthreshold problem drinkers in a population-based survey of young men, and investigated drinking patterns and longitudinal trajectories over 15 months.

**Materials and methods**: Data were collected in the Cohort Study on Substance Use Risk Factors among young Swiss men in their early twenties (n = 4630) at two time points. They completed the 11 criteria DMS-5 AUD and variables related to drinking patterns.

**Results**: Respectively, 23.2 and 23.5 % of the participants were subthreshold problem drinkers at baseline and follow-up. Only 29.4 % of them stayed subthreshold problem drinkers over time (34.1 % becoming AUD drinkers, 36.5 % sympton-free drinkers). AUD status with two criteria was also instable (25.1 % stable over time). Subthreshold problem drinkers showed concurrent and later drinking patterns intermediate between symptom-free and AUD drinkers.

**Conclusions**: Subthreshold problem drinkers were an important subgroup of drinkers with risky drinking patterns, but they do not necessarily progress to a later AUD status and do not appear as a consistent subgroup over time. Thus, the current DSM-5 classification appeared to better capture less severe forms of AUD. However, the instability of AUD status with two criteria questions the cutoff of two criteria. A cutoff of three should be appropriate to define AUD, at least for screening in population-based survey of young people.

## A10 **Motivation-based and skill-based**: **a framework for characterizing common factor processes in brief interventions for behavior change**

### Molly Magill

#### Center for Alcohol and Addiction Studies, Brown University, Providence, RD, USA

**Correspondence:** Molly Magill - molly_magill@brown.edu

*Addiction Science & Clinical Practice* 2016, **11(Suppl 1)**: A10

**Background**: The debate surrounding *common* versus *modality*-*specific* processes of change is long-standing. In the absence of differential efficacy between evidence-based brief interventions, tests for modality-specific mechanisms are compromised. Yet, characterizing all brief interventions by a single process model could be overly simplistic.

**Objective**: This presentation argues many brief interventions can be characterized by differential reliance on *motivation*- and *skill*-*based* methods.

**Materials and methods**: This study examined three classes of therapeutic behaviors (*Exploring* attitudes about behavior change; *Teaching* about coping behaviors; and *Connecting* to clients) and their relationship to client change language about *proximal* (i.e., coping behaviors) and *distal* (i.e., alcohol use) change (change talk [CT]). Two novel observational coding systems (Magill & Apodaca 2011a, b) were applied to three treatments for alcohol use disorder (Cognitive-Behavioral Therapy [CBT]; Twelve-Step Facilitation [TSF]; Motivational Enhancement Therapy [MET]). Sequences of coded behaviors were entered into GSEQ, and transitional probabilities were computed for four sessions. Treatment condition was treated as a moderator of transition probability magnitude in subsequent analyses.

**Results**: Compared to MET, CBT and TSF were less likely to elicit drinking- (*B*_*CBT*_ = −.08, *SE* = .01, *p* < .001; *B*_*TSF*_ = −.08, *SE* = .01, *p* < .001) and coping-related CT (*B*_*CBT*_ = −.04, *SE* = .01, *p* = .001; *B*_*TSF*_ = −.04, SE = .01, p = .002) from exploratory behaviors. TSF therapists, but not CBT therapists, were less likely than MET therapists to elicit drinking-related CT (*B* = −.03, *SE* = .01*, p* = .001) and more likely to elicit coping-related CT (*B* = .03, *SE* = .01*, p* = .028) from teaching behaviors. Transitions from connecting behaviors to CT did not vary by condition.

**Conclusions**: The analyses illustrate partial support for the argument that CBT and TSF differ from MET primarily via differential reliance on motivation- versus skill-based interventions.

## A11 Access to harm-reduction interventions tailored to socially marginalized individuals with a history of substance use in a drop-in center

### Caroline Graap^1^, Sophie Paroz^1^, Véronique S. Grazioli^1^, Susan E. Collins^2^, Jean-Bernard Daeppen^1^

#### Alcohol Treatment Center, Lausanne, Lausanne University Hospital, Lausanne, 1011, Switzerland; ^2^University of Washington-Harborview Medical Center, Seattle, WA 98195, USA

**Correspondence:** Caroline Graap - caroline.graap@chuv.ch

*Addiction Science & Clinical Practice* 2016, **11(Suppl 1)**: A11

**Background:** Despite their needs of treatment, socially marginalized individuals with a history of substance use experience difficulties in accessing traditional healthcare. Developing person-centered treatment tailored to their specific needs and expectations is therefore important. A harm-reduction drop-in center allowing alcohol consumption onsite has recently opened in Lausanne, Switzerland. Harm-reduction interventions were provided to interested attendees through a partnership with the Alcohol Treatment Unit, Lausanne University Hospital.

**Materials and methods**: Drop-in center attendees were not required to attend treatment. A psychologist was however available onsite to provide interested attendees with services. Interventions were free of charge, anonymous, and appointments were optional. Discussed problematic and types of interventions were monitored during an 18-month pilot phase.

**Results**: Seventy-one individuals (17.8 % of the drop-in attendees, 87.3 % men) received at least one intervention and 220 interventions were monitored over the pilot phase. Content analysis yielded three intervention categories: (a) brief intervention (e.g., harm reduction, psychosocial support), (b) counseling sessions (e.g. psychological support, psychotherapy) and (c) liaison activities. (e.g. accompaniment to appointments). Alcohol use was the most discussed problematic followed by health-related issues, access to social and healthcare network, psychosocial situation and other substance use. Number of interventions ranged from a single intervention to long-term support. Finally, an increasing number of attendees came spontaneously to the psychologist and attendees showed a growing interest for meetings without appointment over time.

**Conclusions**: Findings indicated that drop-in center attendees had various demands and were interested in receiving harm-reduction brief interventions and/or counseling. Increased number of spontaneous demands over time suggests that offering onsite psychologist’s services to interested attendees may fit this population’s expectancies. These preliminary findings suggest that socially marginalized individuals show concern for their substance use and their health and are interested in receiving person-centered interventions. Further evaluation should examine whether these interventions are related to substance use and quality-of-life outcomes.

## A12 **An economic and health assessment of a brief intervention for adolescents with problematic substance use**: **10** **year outcomes**

### Robert J. Tait^1^, Lucinda Teoh^2,3^, Erin Kelty^2^, Elizabeth Geelhoed^3^, David Mountain^4, 5^, Gary K. Hulse^2^

#### ^1^National Drug Research Institute, Faculty Health Science, Curtin University, Perth, Western Australia, 6102, Australia; ^2^School of Psychiatry and Clinical Neurosciences, the University of Western Australia, Perth, Western Australia, 6009, Australia; ^3^School of Population Health, the University of Western Australia, Perth, Western Australia, 6009, Australia; ^4^School of Primary, Aboriginal & Rural Health Care, the University of Western Australia, Perth, Western Australia, 6009, Australia; ^5^Department of Emergency Medicine, Sir Charles Gairdner Hospital, Perth, Western Australia, 6009, Australia

**Correspondence:** Robert J. Tait - robert.tait@curtin.edu.au

*Addiction Science & Clinical Practice* 2016, **11(Suppl 1)**: A12

**Background**: The use of alcohol or other drugs (AOD) is prevalent among adolescents in Australia and results in significant costs to society and disease burden to young people. The effectiveness and practicality of brief interventions (BI) delivered in hospital emergency departments (ED) to address this issue remains in doubt.

**Materials and methods**: In 1999–2002, we coordinated a randomized trial in four metropolitan ED in Perth, Western Australia. Participants were adolescents aged 12–19 years with an AOD-related presentation. We compared usual care (n = 67) with a BI (n = 60) that facilitated access to external AOD services. We have now used the West Australian data Linkage System to compile records on health outcomes, especially the use of ED services over the subsequent 10 years. From these data we estimated rates of events and costs. Highly skewed data were winsorised before analyses using generalized linear models.

**Results**: Overall, there were 441 and 479 ED presentations by the BI and control group respectively, which involved 49 people from each group. The BI had a significant impact on both the mean costs ($22 vs. $227: z = 3.16, p = 0.002) and rates (0.03 vs. 0.25: z = 2.79, p = 0.005) of ED mental health AOD presentations (2 vs. 41). However, there was no significant difference in the overall costs ($4266 vs. $4150, p = 0.916) or rates (6.8 vs. 6.5 p = 0.838) associated with all ED presentations.

**Conclusions**: A BI targeting AOD use by adolescents and facilitating their access to AOD services can have lasting impacts on their health outcomes as assessed in terms of ED events related to AOD consumption. However, this did not generalize to other adverse health outcomes that result in ED presentations.

*Trial registration*: The trial was initiated (1999) before registration became standard practice

## A13 Social workers’ and their clients’ attitudes toward alcohol-related problems

### Elina Renko

#### Department of Social Sciences, University of Helsinki, Helsinki, 00170, Finland

**Correspondence:** Elina Renko - elina.renko@helsinki.fi

*Addiction Science & Clinical Practice* 2016, **11(Suppl 1)**: A13

**Background:** Alcohol-related problems are widely viewed as health problems. This view means that identifying and managing alcohol-related problems can be seen as marginal to the social workers’ job. However, social workers frequently encounter alcohol-related problems in the course of their practice. This study explores the topic and presents a qualitative analysis of social workers’ and their clients’ attitudes toward alcohol-related problems.

**Materials and methods:** The study employs a qualitative attitude approach. The aim of the approach is to explore the construction of attitudes in argumentative talk. Social workers (N = 14) and their clients (N = 14) were asked to comment on the eight statements concerning identification and management of alcohol-related problems. The primary objective is to explore how alcohol-related problem was constructed as an attitude object. The analytical focus is on how the two parties constructed alcohol-related problem in their arguments and did workers and customers do this in a same way or were there differences between them?

**Results:** Analysis of the qualitative data reveals that both groups mainly constructed alcohol-related problem as a social issue. The interviewees associated this social issue closely with social statuses as well as with client’s fulfillment of their responsibilities, and their ability to function well. Alcohol-related problem was allocated not only to the individual but to people around him as well. The medicalized view of alcohol-related problem—highlighting the negative impact this problem can have on people’s health and well-being—was also present in the argumentative talk but was less common than the social view.

**Conclusions:** Both the social workers and their clients acknowledged alcohol-related problems as a social issue and not just a health issue. Thus, they saw identifying and managing alcohol-related problems as essential to the social workers’ job. This social view might be in contrast with the individualistic models of substance abuse treatment.

## A14 Application of system dynamics to inform a model of adolescent SBIRT implementation in primary care settings

### Shannon G. Mitchell^1^, David Lounsbury^2^, Zhi Li^3^, Robert P. Schwartz^1^, Jan Gryczynski^1^, Arethusa S. Kirk^4^, Marla Oros^5^, Colleen Hosler^5^, Kristi Dusek^1^, Barry S. Brown^1^

#### ^1^Friends Research Institute, Baltimore, MD, 21201, USA; ^2^Albert Einstein College of Medicine, Yeshiva University, Bronx, NY, 10461, USA; ^3^College of Global Public Health, New York University, New York, NY, 10003, USA; ^4^United Health Care, Baltimore, MD, 21045, USA; ^5^The Mosaic Group, Baltimore, MD, 21210, USA

**Correspondence:** Shannon G. Mitchell - smitchell@friendsresearch.org

*Addiction Science & Clinical Practice* 2016, **11(Suppl 1)**: A14

**Background**: The implementation of screening and brief intervention within primary care settings poses significant challenges related to complexity of the setting as well as the coordination of staff. The proposed presentation will describe the application of system dynamics (SD) modeling to better understand the influence of different implementation strategies on the effective implementation of adolescent screening and brief intervention for substance use in US urban primary care clinics.

**Materials and methods**: Using data from an on-going cluster randomized trial of adolescent SBIRT implementation involving seven federally qualified health center sites we examined the effect of varying quality and frequency of training and trouble-shooting efforts. Simulated over a 20-month intervention implementation period, we used our SD model to compare our ‘Basecase’ (calibrated) outcome [i.e., High quality on-going technical assistance (TA) with quarterly site-specific performance feedback reporting (PFR)] to five strategy scenarios.

**Results**: Our SD model, supported by qualitative and quantitative data from the study, effectively represented the SBIRT intervention, which was calibrated to reflect actual monthly volume of adolescent primary care visits (N = 10,090), screenings (N = 5452), positive screenings (N = 1363), and brief interventions (BIs; N = 49). Decreasing PFR to twice per year (Bi-annual) as opposed to quarterly, and decreasing quality of TA by 50 % served to reduce BI delivery by two-thirds (S1 and S4 64.7 % and 68.1 % reduction, respectively, by month 20). Merely reducing the quality of TA by 25 % was least detrimental (S2; 36.2 % reduction by month 20). Most detrimental to BI delivery were reductions in both TA and PFR (S3 and S5 78.5 % and 89.6 % reduction, respectively, by month 20).

**Conclusions**: SD modeling is a robust method for comparative analyses of implementation strategies. This approach facilitates synthesis of multiple sources of information/data and can foster important insights about how to deploy limited resources for training and support in diverse clinical sites.

*Trial registration*: NCT01829308

## A15 **Drowning in data**: **7500 responses to a text message intervention**

### Iain K. Crombie^1^, Linda Irvine^1^, Brian Williams^2^, Falko F. Sniehotta^3^, Ambrose Melson^4^

#### ^1^Division of Population Health Sciences, University of Dundee, Dundee, DD2 4BF, UK; ^2^Nursing, Midwifery & Allied Health Professions, University of Stirling, Stirling, FK9 4LA, UK; ^3^Institute of Health and Society, Newcastle University, Newcastle upon Tyne, Tyne and Wear, NE1 7RU, UK

**Correspondence:** Iain K Crombie - i.k.crombie@dundee.ac.uk

*Addiction Science & Clinical Practice* 2016, **11(Suppl 1)**: A15

**Background**: Text message interventions are increasingly being used to modify adverse health behaviors. However there are no established techniques for writing effective text message interventions. This study aims to identify the characteristics of text messages that effectively engaged disadvantaged men in an alcohol intervention.

**Materials and methods**: 825 men from disadvantaged areas were recruited to an intervention to reduce the frequency of binge drinking. The 413 men in the intervention group received 112 text messages, 21 of which prompted a response.

**Results**: Just over 7500 responses were received from the intervention group. The number of responses to the intervention texts which prompted a reply ranged from 101 to 340 with an average of 222 responses. The prompts covered key steps in the behavior change model such as: self-monitoring of drinking (266 responses); problems caused by drinking (309 responses); reasons for cutting down (318 responses); goal setting (195 responses); action planning (214 responses). Often men give detailed sensitive personal information about their drinking and the harms it causes them and their families. They also describe their attempts at drinking less, the setbacks encountered and the benefits they enjoy when they are successful at cutting down.

**Conclusions**: Text messages elicit real-time responses which give insight into the key steps in the behavior change process. The characteristics of texts which prompt most responses identify the techniques for developing effective text message interventions.

*Trial registration*: ISRCTN07695192

## A16 Integrating substance use-related screening, brief intervention and referral to treatment in prelicensure nursing curricula

### Deborah S. Finnell^1^, Aisha Holloway^2^

#### ^1^School of Nursing, Johns Hopkins University, Baltimore, MD, 21218, USA; ^2^Nursing Studies, School of Health in Social Science, The University of Edinburgh, Edinburgh, EH8 9AG, UK

**Correspondence:** address: Deborah S. Finnell - dfinnel1@jhu.edu

*Addiction Science & Clinical Practice* 2016, **11(Suppl 1)**: A16

**Background**: Adding content to nursing curricula is often met with resistance, given the required content that must be included to prepare students for the registered nurse role. Globally, the evidence suggests that alcohol education is lacking within nursing undergraduate curricula. The purpose of this presentation is to illustrate how a range of alcohol-related education and training module content can be integrated into the baccalaureate curriculum in two Schools of Nursing within large universities in the US and UK.

**Materials and methods**: Supported by funding from the US Substance Abuse and Mental Health Services Administration (SAMHSA), five core modules (SBIRT Overview, Screening, Motivational Interviewing, Brief Intervention, Referral to Treatment), four substance-related modules (Prevention of Harm, Transtheoretical Model, Neurobiology Underlying Substance Use, Pharmacotherapy for Substance Use Disorders), four clinically-focused specialty modules (Withdrawal and Detoxification, Pain and Opioids, Older Adults, Adolescents), and one module on implementing SBIRT in practice were developed. A 4-step process for planning and implementing the curriculum guided the work: laying the groundwork, adapting the content to the curriculum, implementing, and refining.

**Results**: Curricular maps for Johns Hopkins School of Nursing (US) and Nursing Studies, University of Edinburgh (UK) were developed to guide the placement and timing of the content for delivery to nursing students who will be eligible for licensure as registered nurses. Rather than placing all content in a stand-alone course, integrating content in courses resulted in a threefold increase from the current exposure for nursing students in the US. The model is currently being translated for implementation at the University of Edinburgh.

**Conclusions**: Approximately 9 h of specialty substance-use related SBIRT content can be delivered by integrating the content into existing courses across a prelicensure nursing curriculum. The proposed implementation model can be utilized by nurse educators wishing to provide an optimal integrative program of alcohol education.

## A17 Validation of the tobacco, alcohol, prescription medication, and other substance use (TAPS) tool for identification of problem use and substance use disorders in U.S. primary care patients

### Jennifer McNeely^1^, Li-Tzy Wu^2^, Geetha Subramaniam^3^, Gaurav Sharma^4^, Robert P. Schwartz^5^

#### ^1^Department of Population Health, New York University School of Medicine, New York, NY, 10016, USA; ^2^Department of Psychiatry and Behavioral Sciences, Department of Medicine and Duke Clinical Research Institute, Duke University Medical Center, Durham, NC, 27710, USA; ^3^Center for the Clinical Trials Network, National Institute on Drug Abuse, National Institutes of Health, Bethesda, MD, 20892, USA; ^4^The EMMES Corporation, Rockville, MD, 20850, USA; ^5^Friends Research Institute, Inc., Baltimore, MD, 21201, USA

**Correspondence:** Jennifer McNeely - jennifer.mcneely@nyumc.org

*Addiction Science & Clinical Practice* 2016, **11(Suppl 1)**: A17

**Background**: The TAPS Tool was developed to provide a substance use screening approach that is brief, accurate, and sufficiently detailed to inform clinical care in medical settings. Through a 4-item screen followed by a modified ASSIST-lite, it identifies past-year use of tobacco, alcohol, illicit drugs, and non-medical use of prescription medications, and provides substance-specific assessment of current use and risk level for eight substance classes. This study sought to validate the TAPS Tool in primary care patients.

**Materials and methods**: A multi-site validation study was conducted by the National Drug Abuse Treatment Clinical Trials Network in five geographically diverse primary care clinics. A total of 2000 adults were consecutively enrolled from clinic waiting areas. Participants were randomly assigned in counter-balanced order to complete interviewer-administered and self-administered (on an iPad) versions of the TAPS Tool. The TAPS Tool was compared to the reference standard modified Composite International Diagnostic Interview to determine its diagnostic accuracy for identifying current problem use and DSM-5 substance use disorder (SUD) for each substance class.

**Results**: The self-administered and interviewer-administered TAPS Tool had similar diagnostic characteristics. For identifying problem use, at a cutoff score of >1 the TAPS Tool had sensitivity and specificity of 0.93 and 0.87, respectively, for tobacco, and 0.74 and 0.79 for alcohol. For problem use of illicit and prescription drugs, sensitivity ranged from 0.82 for marijuana to 0.63 for sedatives, and specificity was 0.93–1.0. For identifying SUD, at a cutoff of >2, sensitivity of the TAPS Tool ranged from 0.74 for tobacco to 0.48 for prescription opioids, and specificities were 0.89 or greater.

**Conclusions**: The TAPS Tool detected clinically relevant substance use and risk level in a diverse sample of primary care patients, and could ease barriers to incorporating substance use screening into medical settings.

*Trial registration*: ClinicalTrials.gov # NCT 02110693

## A18 **Treatment of alcohol dependence**: **a randomized controlled trial comparing treatment in primary care with specialized addiction treatment**

### Sara Wallhed Finn, Sven Andreasson

#### Department of Public Health Sciences, Karolinska Institutet, Stockholm, 171 77, Sweden

**Correspondence:** Sara Wallhed Finn - sara.wallhed-finn@sll.se

*Addiction Science & Clinical Practice* 2016, **11(Suppl 1)**: A18

**Background**: Alcohol dependence has the largest treatment gap between the number of people affected and the number in treatment, of all psychiatric disorders. While there is good evidence that treatment is effective, only one in ten in Sweden are reached by the present treatment system. There are several reasons for not seeking treatment, one is the stigma attached to drinking problems and treatment seeking. A possible alternative approach, with a lower degree of stigma, involves a greater role for primary care (PC) in the treatment of alcohol dependence. There are a number of treatment methods that can be applied by generalists in PC.

The aim is to evaluate whether a new form of treatment for alcohol dependence in PC is equally effective as treatment in specialist treatment in a dependence clinic.

**Materials and methods**: 288 persons with alcohol dependence have been recruited through media advertisements, and randomly assigned to treatment either at a specialized addiction clinic or at a PC unit. Treatment at the specialist clinic is treatment as usual, including pharmacological and/or psychosocial programs. GPs at 12 PC units have been trained for 8 h in a brief treatment program, the 15-method. In this program, patients are in the first session offered feedback on their baseline assessment and advice. Patients requesting more treatment are offered three brief advice sessions together with pharmacological treatment. Primary outcome in this study is alcohol consumption. Secondary outcomes include severity of dependence, consequences of drinking, psychological health, quality of life and biomarkers.

**Results**: At 6 months 232 participants were followed up (81 %). Preliminary results indicate no statistically significant differences in outcome between the two study alternatives

**Conclusions**: If these results hold up, they suggest that alcohol dependence can be successfully treated by general practitioners in primary care.

*Trial registration*: Trial identifier ISRCTN84490505 at Controlled Trials.com

## A19 Development of a web-based deviance regulation intervention to increase protective behavioral strategies during spring break

### Robert D. Dvorak^1^, Matthew P. Kramer^1^, Brittany L. Stevenson^1^, Emily M. Sargent^2^, Tess M. Kilwein^2^

#### ^1^Department of Psychology, University of Central Florida, Orlando, Florida, 32816, USA; ^2^Department of Psychology, University of North Dakota, Grand Forks, North Dakota, 58202, USA

**Correspondence:** Robert D. Dvorak - rob.d.dvorak@gmail.com

*Addiction Science & Clinical Practice* 2016, **11(Suppl 1)**: A19

**Background**: Spring break (SB) is an annual vacation for college students. SB vacations are often associated with heavy episodic drinking and increased alcohol-related risks. This may be especially relevant for women. The current study utilized Deviance Regulation Theory (DRT) to increase the use of protective behavioral strategies (PBS) among female college students on SB.

**Materials and methods**: Female college students going on SB (n = 62) completed a screening, a Pre-SB intervention (where they were randomly assigned to receive either a positively or negatively framed message about individuals who do or do not use PBS), and a Post-SB assessment that provided alcohol and PBS use data for each day of SB (n = 620 person days).

**Results**: Data was analyzed using a multilevel structural equation model. PBS use during SB increased as SB PBS norms became more normative in the negative frame condition (B = −0.023, p = .006). In the positive frame condition, PBS use during SB increased as SB PBS use norms became less normative (B = 0.034, p < .001). These associations did not result in lower alcohol consumption, but did result in lower alcohol-related problems during SB through PBS use for both the positive (p = .026) and negative (p = .037) frames.

**Conclusions**: This data suggests that a simple online intervention could be used to quickly tailor and deliver a salient message about individuals who do or do not engage in responsible drinking patterns. This tailored message results in more responsible drinking and fewer alcohol-related problems. Given the ease of implementation and the significant problems associated with SB, this intervention could have a substantial public health impact.

## A20 Reliability and validity of past-12-month use frequency items as opening questions for the CRAFFT adolescent substance abuse screening system

### Sion K. Harris^1,2^, Lon Sherritt^1,2^, Sarah Copelas^1^, John R. Knight, Jr.^1,2^, The Partnership for Adolescent Substance Use Research

#### ^1^Department of Pediatrics, Harvard Medical School, Boston, MA, 02115, USA; ^2^Center for Adolescent Substance Abuse Research, Boston Children’s Hospital, Boston, MA, 02115, USA

**Correspondence:** Sion K. Harris - sion.harris@childrens.harvard.edu

*Addiction Science & Clinical Practice* 2016, **11(Suppl 1)**: A20

**Background**: To obtain consumption information, the “yes”/“no” opening questions to the CRAFFT, a widely-used adolescent substance abuse screener, were replaced with questions asking for past-12-month number of substance use days. The aim of this study was to assess the reliability and criterion validity of these consumption opening questions among adolescent primary care patients.

**Materials and methods**: We recruited an age-gender-balanced convenience sample of 708 12- to 18-year-old patients arriving for annual well-visits from February 2015-April 2016 at five pediatric primary care offices in Massachusetts USA. Before the medical visit, participants self-administered the screener on a tablet computer. The opening items asked “During the past 12 months, on how many days did you use [alcohol, cannabis, etc.]?”, with numeric key-pad response and instruction “(Put 0 if none)”, followed by the “Car” item of the CRAFFT. Only those reporting any use days subsequently completed the five RAFFT questions (Relax, Alone, Forget, Family/Friends, Trouble). A confidential research staff-administered 12-month substance use Timeline Follow-Back (TLFB) interview was the criterion standard. For test–retest reliability, a subsample of 250 participants completed the identical screener within 2 weeks. We computed sensitivity (Sn), specificity (Sp), positive/negative likelihood ratios (±LR) for any-use validity. Intra-class correlation coefficients (ICC) were computed for days-of-use validity and test–retest reliability (95 % confidence intervals for all). All participating IRBs approved the study protocol, including a waiver of parental consent.

**Results**: Compared to the criterion standard, the screener’s consumption items had Sn = .96 (.94–.97), Sp = .81 (.74–.86), LR+ = 5.0 (3.7–6.9) LR− = .05 (.04–.08) for identifying any use. ICCs for days-of-use were alcohol = .79 (.76–.81) and marijuana = .84 (.81–.86). There was insufficient other-drug use for analysis. Test–retest reliability was high: ICCs for alcohol = .84 (.80–.88) and marijuana = .88 (.85–.90).

**Conclusions**: Asking adolescents to input the number of past-12-month days of use is a valid and reliable way to initiate CRAFFT screening.

## A21 Scoping review to develop a community pharmacy-based screening and brief intervention for unhealthy alcohol use in the UK

### Noreen D. Mdege, Jim McCambridge

#### Department of Health Sciences, University of York, York, YO10 5DD, UK

**Correspondence:** Noreen D Mdege - noreen.mdege@york.ac.uk

*Addiction Science & Clinical Practice* 2016, **11(Suppl 1)**: A21

**Background**: Community pharmacies have the potential to make a significant contribution to dealing with unhealthy alcohol use. Evidence is lacking, however, on the nature of the interventions appropriate for delivery in this setting. This scoping review was carried out in-order to gather evidence to inform the development of an intervention that is appropriate for identifying and intervening with unhealthy alcohol users within community pharmacies in the UK.

**Materials and methods**: This was a review of international empirical evidence, and UK policy and pharmacy profession documents to address the following questions:

1. What is the effectiveness of community pharmacy-based public health interventions such as those for alcohol consumption?
2. What are the challenges to the delivery of such interventions within community pharmacies?
3. What is the current UK policy on the delivery of such interventions within this setting?
4. What are the pharmacy profession and general public’s views on the role of community pharmacies in providing such interventions?

**Results**: In the UK the provision of pharmacy-based public health interventions such as those for alcohol consumption is a national priority, and is defined as one of the core pharmacist’s roles. However, evidence on the effectiveness of delivering such interventions within community pharmacies is largely inconclusive. That on interventions for alcohol use in particular is very much underdeveloped. Although pharmacists are very willing to deliver screening and brief interventions for unhealthy alcohol use, they lack the skills and competencies needed to do this effectively. There is also uncertainty among the general public on whether the pharmacist is qualified to provide these interventions.

**Conclusions**: The expectation for community pharmacies to provide standalone behavior change interventions for unhealthy alcohol use may be problematic. There is need to consider their involvement in identification of unhealthy alcohol users and referral to care pathways where intervention effectiveness evidence is more established.

## A22 What predicts treatment entry in proactively recruited individuals with DSM-5 Alcohol Use Disorder?

### Gallus Bischof^1^, Anja Bischof^1^, Jennis Freyer-Adam^2^, Hans-Juergen Rumpf^1^

#### ^1^Department of Psychiatry, University of Luebeck, Luebeck, 23538, Germany; ^2^Institute of Epidemiology and Social Medicine, University of Greifswald, Greifswald, 17489, Germany

**Correspondence:** Gallus Bischof - gallus.bischof@uksh.de

*Addiction Science & Clinical Practice* 2016, **11(Suppl 1)**: A22

**Background**: Although Screening and Brief Interventions have proven efficacy in individuals with at-risk drinking in the absence of alcohol use disorders (AUDs), addressing the more severely affected individuals remains challenging. Brief Interventions (BI) promoting treatment entry have not yet proven efficacious. The present exploratory analysis aims to identify predictors of treatment entry in individuals with AUDs according to DSM-5 recruited in general practices and general hospitals.

**Materials and methods**: Participants were recruited in general practices and wards of two general hospitals as part of the interventional RCTs “Stepped-care Intervention for Problem drinking” SIP and “Expert-system intervention for problematic Alcohol use” ExtrA via systematic screening. Eligible participants were diagnosed using the Munich Composite International Diagnostic Inventory (M-CIDI). Among 756 participants included in the studies, 493 Participants were holding an AUD according to DSM-5. Among these, 81 subjects received treatment for AUDs within a 12-month follow-up. AUD-related and motivational variables were assessed using standardized instruments at baseline and were analyzed using logistic regression to predict treatment entry.

**Results**: Individuals who received treatment during the follow-up period revealed higher reductions in drinking in relation to their intake at baseline compared to non-treated individuals. They revealed higher problem severity, lower mental health, lower health status, higher motivation to change and higher readiness to enter alcohol-related treatment at baseline. In a stepwise multivariate logistic regression controlling for recruitment setting, statistically significant predictors of treatment entry were readiness to change according to the TTM and readiness to seek treatment, irrespective of having received BI.

**Conclusions**: Data suggest that motivational factors that might be addressed by BI are a crucial issue for promoting treatment entry. Further research on motivational interventions fostering treatment entry of individuals with AUDs is needed and appears promising.

*Trial registration*: SIP: ClinicalTrials.gov Identifier: NCT00391742

*ExtrA*: ClinicalTrials.gov Identifier: NCT00400010

## A23 **Approaches to alcohol screening and brief interventions in antenatal care**: **the conversation matters**

### Niamh Fitzgerald^1^, Lisa Schölin^1^

#### ^1^Institute for Social Marketing, UK Centre for Tobacco and Alcohol Studies, Faculty of Health Sciences and Sport, University of Stirling, Stirling, FK9 4LA, UK

**Correspondence:** Niamh Fitzgerald - niamh.fitzgerald@stir.ac.uk

*Addiction Science & Clinical Practice* 2016, **11(Suppl 1)**: A23

**Background**: Alcohol use in pregnancy is a leading cause of developmental disorders and antenatal care is an important arena for prevention [1]. In Scotland, a high profile, national program of screening and alcohol brief interventions (ABI) was implemented in routine antenatal care as part of a wider Scottish Government initiative [2]. This paper explores the experiences of implementation leaders in local areas who sought to change antenatal practices such that midwives would routinely ask about, identify and respond to alcohol consumption by pregnant women.

**Materials and methods**: A secondary analysis was conducted of 8 semi-structured in-depth interviews, conducted with implementation leaders in 8 (of 14) health administrative areas in Scotland as part of a wider study [3]. Analysis focused on how the screening and intervention process was understood, adapted and implemented in the eight areas and included an examination of documentation provided by interviewees where available. Interviews were transcribed and analyzed thematically.

**Results**: Interviewees reported uncertainty as they tried to implement the national program without clear guidance on what ABIs should consist of in antenatal care. Different areas developed their own models of asking, identifying and responding to consumption. Many rejected formal screening tools and there was no consensus on best practice. Some areas focused on delivering interventions to women who disclosed current alcohol use, although in some areas very few women did so, which was seen as under-reporting. Other health boards focused on pre-pregnancy drinking to try to improve disclosure rates. Two areas reported that, in their experiences, levels of disclosure varied depending on how questions on alcohol use were asked.

**Conclusions**: Local health services in Scotland developed their own tailored approaches to asking about, identifying and responding to alcohol use in pregnant women. More research is needed into the optimal formulation of questions around drinking to support disclosure and intervention where needed.

References

Patra J, Bakker R, Irving H, Jaddoe VWV, Malini S, Rehm J. Dose–response relationship between alcohol consumption before and during pregnancy and the risks of low birthweight, preterm birth and small for gestational age (SGA)-a systematic review and meta-analyses. BJOG. 2011;118(12):1411–21.

Scottish Government. Changing Scotland’s Relationship with Alcohol: a discussion paper on our strategic approach. Edinburgh; 2008.

Fitzgerald N, Platt L, Heywood S, McCambridge J. Large-scale implementation of alcohol brief interventions in new settings in Scotland: a qualitative interview study of a national programme. BMC Public Health. 2015;15(1):289.

## A24 A systematic review of alcohol screening and assessment measures for young people

### Paul Toner^1^, Jan R. Böhnke^1,2^, Jim McCambridge^1^

#### ^1^Department of Health Sciences, University of York, York, Yorkshire, YO10 5DD, UK; ^2^Hull York Medical School, University of York, York, Yorkshire, YO10 5DD, UK

**Correspondence:** Paul Toner - paul.toner@york.ac.uk

*Addiction Science & Clinical Practice* 2016, **11(Suppl 1)**: A24

**Background**: This systematic review evaluates the validity of available instruments for screening and assessing alcohol consumption and related problems in young people aged 24 and under. Highlighting the best performing assessment tools which correctly identify young people at risk from alcohol-related harm provides researchers, practitioners and policy makers with the means of measuring prevalence and patterns of risk and can inform appropriate interventions.

**Materials and methods**: A systematic review and narrative synthesis of studies reporting instrument properties from six electronic databases (including EMBASE, MEDLINE, PsycINFO, Social Sciences Citation Index) containing both peer-reviewed and grey literature. The review was conducted in accordance with the Centre for Reviews and Dissemination (CRD) guidance (CRD 2009). A modified COSMIN checklist was used to analyse the quality of psychometric studies for instruments achieving good levels (above 0.7) of reliability and predictive validity.

**Results**: From the initial search 55 measures have been identified assessing alcohol use or substance use (with alcohol-related items) and/or associated problems. The review identified 26 instruments designed specifically for young people. Other instruments included modified adult measures or adult measures administered to youth populations. Preliminary findings indicate particular issues with the reliability of items used to capture alcohol-related consequences including dependence for young people. Screening tools also warrant further validation studies.

**Conclusions**: This study provides a synthesis of validation data for the best performing alcohol screening and assessment measures for young people. Further work will be required to adapt and develop reliable and valid alcohol tools for the youth population.

## A25 **Alcohol interventions**: **A randomized study examining two brief counseling interventions**

### Laura J. Veach, Olivia Currin, Leigh Z. Dongre, Preston R. Miller, Elizabeth White

#### General Surgery, Wake Forest School of Medicine, Winston-Salem, NC, 27517, USA

**Correspondence:** Laura J. Veach - lveach@wakehealth.edu

*Addiction Science & Clinical Practice* 2016, **11(Suppl 1)**: A25

**Background**: The aim of the presentation reviews two brief counseling intervention (BCI) findings. One, a personalized BCI (PBCI), included key components of engagement, contributors to drunkenness, and changes in drinking patterns.

**Materials and methods**: Eligible trauma patients were randomized to one of two BCIs. One BCI, NIAAA-recommended quantity and frequency of consumption (NIAAA 2008) or another, a PBCI explored qualitative experiences of drunkenness, including perceptions of overdoing it and alternatives. Mean baseline drinking behaviors between the BCI groups and changes from baseline to 6 months were compared.

**Results**: Of the 507 eligible patients, 333 were consented; 181 participants completed follow-up at 6 months. The average BCI length was significantly higher (t = 2.2252, df = 329, p = 0.0267) in the personalized BCI (31 min) compared to the quantitative BCI (27.5 min). There was no statistical difference in self-reported satisfaction between the two intervention groups; both groups indicated strong satisfaction. There was no statistical difference in changes in drinking at the follow-up between the BCI groups. Both BI groups showed significantly fewer typical drinks consumed and decreased alcohol tolerance at follow-up. In addition, participant engagement was examined using measured change between pre-BI and post-BI scores as rated by BCI counselors. The scale was developed based on previous findings utilizing similar items (e.g., Magill et al. 2010; Schermer et al. 2006). Participant engagement score analysis suggests that there was no statistically significant difference (p < .05). These results are noteworthy insofar as they suggest that the PBCI may be as efficacious at a conventional quantity/frequency-focused intervention.

**Conclusions**: The results of the current study suggest that perhaps the ubiquitous focus on quantity and frequency of alcohol use is not the only means by which to facilitate changes in drinking behaviors. Results suggest that counselors can provide a personalized approach as an efficacious intervention model.

*Trial registration*: Human subjects review and study oversight were provided by the Wake Forest University Health Sciences (WFUHS) Institutional Review Board (IRB).

The clinical trial was also registered with the National Institute of Health clinicaltrials.gov registry (Identifier: NCT00865774).

## A26 Documented brief intervention not associated with resolution of unhealthy alcohol use at follow-up screening among VA Patients with HIV

### Emily C. Williams^1,2,3^, Gwen T. Lapham^1^, Jennifer J. Bobb^1^, Anna D. Rubinsky^4^, Sheryl L. Catz^5^, Susan Shortreed^1,2^, Kara M. Bensley^2,3^, Katharine A. Bradley^1,2,3^

#### ^1^Group Health Research Institute, Seattle, WA, 98101, USA; ^2^VA HSRD Center of Innovation for Veteran-Centered and Value-Driven Research, Seattle, WA, 98101, USA; ^3^University of Washington, Seattle, WA, 98195, USA; ^4^VA San Francisco, San Francisco, CA, 94121, USA; ^5^University of California at Davis, Sacramento, CA, 95616, USA

**Correspondence:** Emily C. Williams - emily.williams3@va.gov

*Addiction Science & Clinical Practice* 2016, **11(Suppl 1)**: A26

**Background**: Unhealthy alcohol use is particularly risky for HIV+ patients. Brief interventions (BI) reduce drinking among patients with unhealthy alcohol use, but whether receipt of BI in routine outpatient settings is associated with reduced drinking among HIV+ patients with unhealthy alcohol use is unknown. We evaluated this question in a national sample of HIV+ outpatients from the VA Healthcare System.

**Materials and methods**: Secondary VA clinical and administrative data from the electronic medical record (EMR) were used to identify all positive alcohol screens (AUDIT-C score ≥ 5) documented among HIV + patients (10/01/09–5/30/13) followed by another alcohol screen documented 9–15 months later. Unadjusted and adjusted Poisson regression models assessed the association between BI (advice to reduce drinking or abstain documented in EMR) and resolution of unhealthy alcohol use (follow-up AUDIT-C < 5 with ≥2 point reduction).

**Results**: Among 2101 unique eligible HIV + patients with unhealthy alcohol use (2803 records), 77 % of positive screens were followed by documented BI, and 61 % of screens were followed by a follow-up screen reflecting resolved unhealthy alcohol use. Documented BI after a positive screen was not associated with resolution of unhealthy alcohol use at follow-up in unadjusted [Incidence Rate Ratio (IRR) 0.96, 95 % Confidence Interval (CI) 0.90–1.03, p = 0.230] or adjusted analyses (IRR 0.96, 95 % CI 0.90–1.02, p = 0.208).

**Conclusions**: Documented BI was not associated with resolving unhealthy alcohol use at follow-up screening among HIV + outpatients with unhealthy alcohol use. Research is needed to identify effective methods of reducing drinking in this vulnerable sub-population of patients.

## A27 **Making electronic interventions engaging**: **Development of a smartphone app targeting harmful drinking in young adults**

### Joanna Milward^1^, Paolo Deluca^1^, Zarnie Khadjesari^1^, Rod Watson^2^, Stephanie Fincham-Campbell^1^, Colin Drummond^1^

#### ^1^Addictions Department, Institute of Psychiatry, London, SE58BB, UK; ^2^Health Innovation Network, London, SE1 9BB, UK

**Correspondence:** Joanna Milward - joanna.milward@kcl.ac.uk

*Addiction Science & Clinical Practice* 2016, **11(Suppl 1)**: A27

**Background**: Electronic screening and brief intervention (eSBI) is effective in reducing weekly alcohol consumption. Smartphone applications (apps) demonstrate promise in delivering eSBI, however barriers remain in how to optimally engage users with intervention features. The Alcohol Theme of the Collaboration for Leadership in applied Health Research and Care (CLAHRC) South London, is developing an eSBI app targeting harmful drinking in young adults, utilizing a user-centered design (UCD) approach, and the developmental processes are presented here.

**Materials and methods**: Stage 1 of the design process involved focus groups with the target population, determining their preferences for content, features and style. In stage 2 app components were defined utilizing Stage 1 findings, behavior change methods and engagement theory. The research and programming team developed the app in an iterative process in Stage 3, with feedback from the target population. Stage 4 involved final user-testing of the app.

**Results**: The app includes core eSBI functions as well as additional features targeting engagement. Core functions include a drinks diary, goal setting and information on drinking risks and advice for cutting down. Additional engagement promoting strategies for targeting young adults have been developed including ‘gamification’, ‘community’ and ‘tailoring’ features.

**Conclusions**: The app is still under evaluation in an implementation trial, and a pilot randomized controlled trial examining the effectiveness of the engagement strategies. However, work so far suggests that when designing an eSBI app, to optimize engagement, all features of the app should be tailored to the preferences, needs and user habits of the target population.

## A28 **Reported training in alcohol brief intervention trials**: **a systematic narrative synthesis**

### Niamh Fitzgerald^1^, Kathryn Angus^1^, Linda Bauld^1^

#### ^1^Institute for Social Marketing, UK Centre for Tobacco and Alcohol Studies, School of Health Sciences, University of Stirling, Stirling, FK9 4LA, UK

**Correspondence:** Niamh Fitzgerald - niamh.fitzgerald@stir.ac.uk

*Addiction Science & Clinical Practice* 2016, **11(Suppl 1)**: A28

**Background**: While alcohol brief interventions (ABIs) delivered in primary care [1] have been efficacious in research trials, implementation in routine practice has proved difficult [2]. Furthermore efficacy may not be maintained outside of trials, possibly due to a loss of fidelity [3]. Amongst other factors, appropriate training and support for practitioners is essential to enable practitioners to deliver ABIs [4]—either in line with the evidence (in frontline practice), or in line with trial protocols (in research studies). This study aimed to ascertain how published trials of ABIs described the training and support provided to practitioners.

**Materials and methods**: A systematic search of the Cochrane Drugs and Alcohol Group’s specialised register was conducted, supplemented by a citation search from relevant Cochrane reviews. Trials of ABIs delivered by frontline healthcare practitioners were included and data extracted using a systematic approach.

**Results**:

- 780 records were identified from the systematic search. After de-duplicating and screening, 71 papers reporting on 53 trials published between 1987 and 2015 in primary care, trauma/emergency and other settings were included in the analysis.
- Most trial reports (55 %) included training duration, for which the overall median was 3 h (mean 4 h), shorter in primary care and trauma/emergency settings, and longer in other community settings.
- Most trials (62 %) provided no details on the content of training in the published reports.
- Few trials (36 %) reported follow-up support for practitioners following training and fewer reported whether quality checks were made prior to (25 %) or during (25 %) data collection.

**Conclusions**: Practitioner training and support are poorly described in published reports of trials of alcohol brief interventions. Where reported, the nature and intensity of training and support provided is highly variable. Poor reporting impedes analysis of how best to approach the training of frontline health professionals in future ABI research and practice.

References

O’Donnell A, Anderson P, Newbury-Birch D, Schulte B, Schmidt C, Reimer J, Kaner E. The Impact of brief alcohol interventions in primary healthcare: A systematic review of reviews. Alcohol Alcohol. 2013;agt170.

Fitzgerald N, Platt L, Heywood S, McCambridge J. Large-scale implementation of alcohol brief interventions in new settings in Scotland: a qualitative interview study of a national programme. BMC Public Health. 2015;15:289.

Kaner E, Bland M, Cassidy P, Coulton S, Dale V, Deluca P, Gilvarry E, Godfrey C, Heather N, Myles J, Newbury-Birch D, Oyefeso A, Parrott S, Perryman K, Phillips T, Shepherd J, Drummond C. Effectiveness of screening and brief alcohol intervention in primary care (SIPS trial): pragmatic cluster randomised controlled trial. BMJ. 2013;346:e8501.

Fitzgerald N, Molloy H, MacDonald F, McCambridge J. Alcohol brief interventions practice following training for multidisciplinary health and social care teams: A qualitative interview study. Drug Alcohol Rev. 2015;34(2):185–93.

## A29 Do brief alcohol interventions improve self-reported health and mental well-being among general hospital inpatients? 2-year results from the randomized controlled trial PECO

### Jennis Freyer-Adam^1,2^, Sophie Baumann^1,2^, Katja Haberecht^1,2^, Inga Schnuerer^1,2^, Gallus Bischof^3^, Christian Meyer^1,2^, Hans-Jürgen Rumpf^3^, Ulrich John^1,2^, Beate Gaertner^4^

#### ^1^Institute of Social Medicine and Prevention, University Medicine Greifswald, Greifswald, 17489, Germany; ^2^German Center for Cardiovascular Research, Site Greifswald, Greifswald, 17489, Germany; ^3^Department of Psychiatry and Psychotherapy, Medical University of Luebeck, Luebeck, 23562, Germany; ^4^Department of Epidemiology and Health Monitoring, Robert Koch Institute Berlin, Berlin, 13302, Germany

**Correspondence:** Jennis Freyer-Adam - freyer@uni-greifswald.de

*Addiction Science & Clinical Practice *2016, **11(Suppl 1)**: A29

**Background**: Little is known about the long-term effects of brief alcohol interventions (BAI) on measures of health and mental well-being. The aim of this study was to investigate: (1) whether BAIs among at-risk drinking general hospital inpatients improve self-reported health and mental well-being 2 years after initial contact; and (2) whether according intervention effects are dependent on whether BAIs were delivered in-person or through computer-generated feedback letters.

**Materials and methods**: As part of the randomized controlled trial „Testing delivery channels of individualized motivationally tailored alcohol interventions among general hospital inpatients: in-person versus computer-based, PECO” 18–64 year old general hospital inpatients were systematically screened for at-risk alcohol use. Nine-hundred-sixty-one of those who screened positive for at-risk alcohol use and negative for more severe alcohol problems were randomized by timeframe to: a) in-person counseling (PE), b) computer-generated individualized feedback letters (CO) and c) assessment only (AO). Both interventions were designed to include three contacts: on the ward, and 1 and 3 months later. Computer-assisted telephone interviews at months 6, 12, 18 and 24 assessed self-reported health (0, poor—4, excellent) and the 5-item Mental Health Inventory (range: 0–100). Latent growth modeling was used.

**Results**: In comparison to AO, PE and CO resulted in significant improvements of self-reported health at all time-points: With each 6 months, the difference between AO and PE increased by b = 0.05 (p = 0.005) and between AO and CO by b = 0.06 (p = 0.001). In comparison to AO, PE and CO resulted in significant improvements of mental well-being starting in month 12 (PE ΔM = 3.5–5.2, ps < 0.05, CO ΔM = 3.8–7.0, ps < 0.01). PE-CO differences were non-significant.

**Conclusions**: In-person and computer-based BAIs can improve self-reported health and mental well-being among general hospital inpatients, and should be part of routine care.

*Trial registration*: ClinicalTrials.gov: NCT01291693

## A30 **Screening, brief intervention and referral (SBIR) for distress, alcohol and tobacco in an oncology surgical unit**: **qualitative analysis of implementation process and acceptability for patients over one year**

### Marion Barrault-Couchouron^1,6^, Marion Béracochéa^1^, Vincent Allafort^2^, Valérie Barthélémy^2^, Hervé Bonnefoi^2^, Emmanuel Bussières^2^, Véronique Garguil^3^, Marc Auriacombe^3^, Marianne Saint-Jacques^4^, Michel Dorval^5^, Katia M’Bailara^6^

#### ^1^Humanities and social sciences group, Regional Cancer Center Institut Bergonié, Bordeaux, 33076, France; ^2^Oncological Surgery and Oncology Departments, Regional Cancer Center Institut Bergonié, Bordeaux, 33076, France; ^3^Department of Addiction Treatment, Charles Perrens Hospital Center, Bordeaux, 33000, France; ^4^Studies and research programs in addiction diseases, Sherbrooke University, Sherbrooke, Québec, J1 K 2R1, Canada; ^5^Department of Social and Preventive Medicine, Laval University, Québec, G1 V 0A6, Canada; ^6^Laboratory of psychology, University of Bordeaux, Bordeaux, 33076, France

**Correspondence:** Marion Barrault-Couchouron - M.Barrault@bordeaux.unicancer.fr

*Addiction Science & Clinical Practice* 2016, **11(Suppl 1)**: A30

**Background**: Despite evidence-based benefits on reducing morbidity-mortality from cancer, SBIR are poorly implemented in oncology settings. The first objective of this study is to analyze the implementation factors for SBIR alcohol and/or tobacco program, specially adapted for cancer patients, as a routine in an oncology surgical department. The second objective is to assess the acceptability for patients.

**Materials and methods**: Over a year, we conducted and audio-recorded a focus group (N = 5), semi-structured interviews with professionals (N = 3); invited to fill-in an online questionnaire about knowledge and attitudes toward SBIR (N = 25); and collected meeting notes (N = 11). The Consolidated Framework for Implementation Research (CFIR) was used to collect and analyze data. To study patients’ needs and acceptability of the intervention, semi-structured telephone interviews were conducted with randomly selected patients (N = 27). Qualitative triangulation method was use to analyze the data from both perspectives (professional’s and patients).

**Results**: From the providers’ perspective, four domains of the CFIR, were found to be relevant: inner setting, outer setting, characteristics of individuals and intervention. Professionals reported that SBIR conformed to their values (beliefs in quality and effectiveness of intervention). A tense environment and resistance to change were stated as principal barriers to the implementation. In 1 year, 252 patients were screened (26 % were smokers, 20 % presented alcohol use risk and 49 % expressed psychological distress). From patients’ perspective, a high level of participation and satisfaction (92 %) was reported as a result of universal screening.

**Conclusions**: Providing a dedicated time with an identified professional may be a major facilitator of implementation in clinical routine. Acceptability for patients and prevalence of distress and health risk behaviors in our population encourage pursuing implementation efforts to reduce morbidity-mortality from cancer and risk of developing emotional and addictive disorders. However, implementing an SBIR program in an oncology surgical department still remains a challenge.

## A31 Alcohol use problem severity moderates the efficacy of in-person versus computer-based brief alcohol intervention at general hospitals

### Sophie Baumann^1,2^, Beate Gaertner^3^, Katja Haberecht^1,2^, Gallus Bischof^4^, Ulrich John^1,2^, Jennis Freyer-Adam^1,2^

#### ^1^Institute of Social Medicine and Prevention, University Medicine Greifswald, Greifswald, 17489, Germany; ^2^German Centre for Cardiovascular Research, Site Greifswald, 17489, Germany; ^3^Department of Epidemiology and Health Monitoring, Robert Koch Institute Berlin, Berlin, 13302, Germany; ^4^Department of Psychiatry and Psychotherapy, Medical University of Luebeck, Luebeck, 23562, Germany

**Correspondence:** Sophie Baumann - sophie.baumann@uni-greifswald.de

*Addiction Science & Clinical Practice* 2016, **11(Suppl 1)**: A31

**Background**: Evidence on alcohol use problem severity as a moderator of brief alcohol intervention (BAI) efficacy is inconclusive and mostly limited to follow-ups of 12 months or less. The lack of consistent findings may be explained by the heterogeneity in the BAI delivery modality across studies. The aim of this study was to test whether persons with different levels of alcohol use problem severity benefitted differently from in-person versus computer-based BAI over a period of 24 months.

**Materials and methods**: General hospital inpatients with Alcohol Use Disorder Identification Test–Consumption (AUDIT-C) scores ≥4 (for women) or ≥5 (for men) and AUDIT scores < 20 (N = 961, age: 18–64 years) were randomized to in-person counselling (PE), computer-generated individualized feedback letters (CO), or assessment only (AO). PE and CO were delivered at baseline, month 1, and month 3. Outcome was gram alcohol consumed per day after 6, 12, 18, and 24 months. The AUDIT score as an indicator of alcohol use problem severity was tested as a moderator of BAI efficacy.

**Results**: The CO effect was stronger for persons with low AUDIT scores than for those with high scores. At all follow-ups, CO performed significantly better than AO for persons with AUDIT scores below 8 (ps < 0.05). Up to month 18, CO performed significantly better than PE for persons with AUDIT scores below 7–8 (ps < 0.05). Persons with high AUDIT scores tended to benefit more from PE, although PE effects were not statistically significant.

**Conclusions**: Computer-based BAI can be superior over in-person BAI for persons with low levels of alcohol use problem severity. Although our findings indicate that those with higher severity levels may benefit more from in-person BAI, there was a lack of power to detect significant effects among the low number of persons with high AUDIT scores.

*Trial registration*: ClinicalTrials.gov: NCT01291693

## A32 PHC professionals’ knowledge and attitudes towards EIBI on drugs. Results from a survey in Catalonia

### Lidia Segura-Garcia^1^, Nuria Ibañez-Martinez^1^ Juan Manuel Mendive-Arbeloa^2^, Manel Anoro-Perminger^3^, Pako Diaz-Gallego^4^, Mª Angeles Piñar-Mateos^5^, Joan Colom-Farran^1^

#### ^1^Catalonia Department of Health, Public Health Agency, Government of Catalonia, Barcelona, Barcelona, 08005, Spain; ^2^La Mina Primary Health Center, Catalan Institute of Health, Government of Catalonia, Sant Adria de Besos, Barcelona, 08930, Spain; ^3^Besos Primary Health Center, Catalan Institute of Health, Government of Catalonia, Barcelona, Barcelona, 08019, Spain; ^4^Larrard Primary Health Center, PAMEN and Barcelona City Council, Government of Catalonia, Barcelona, Barcelona, 08024, Spain; ^5^Figueres Primary Health Center, Catalan Institute of Health, Government of Catalonia, Figueres, Gerona, 17600, Spain

**Correspondence:** Lidia Segura-Garcia - lidia.segura@gencat.cat

*Addiction Science & Clinical Practice* 2016, **11(Suppl 1)**: A32

**Background**: In Catalonia, less than 1 % of PHC patients are screened for drugs in contrast with 47.1 % for alcohol and 90 % for tobacco. Within the framework of a broader ASSIST feasibility study, a survey was conducted to determine the level of knowledge, skills and attitudes of the PHC professionals towards EIBI on drugs.

**Materials and methods**: A 26-item online survey was developed adapting tools used in previous studies. Main variables included: age, sex, occupation, experience, consultation hours, training on drugs, previous experience with SBI, barriers and facilitators for SBI on drugs and attitudes (adapted SAAPPQ). Survey was promoted among PHC professionals’ associations, PHC specialized websites and tobacco and alcohol SBI networks. It was hypothesized that professionals with previous SBI experience on alcohol and tobacco and with more education on drugs will have better attitudes towards SBI on drugs. Bivariate and multivariate analyses were applied.

**Results**: A total of 752 professionals completed the questionnaire (411 GP and 315 nurses). Lack of training (35.2 %) and time (28 %) were the main implementation barriers. Professionals with previous SBI experience showed significantly higher level of confidence in giving advice on drugs than those without (3.53 vs. 3.31; p = 0.003). In addition, the professionals with more than 10 h of drugs training showed significant differences on role security (16.93 vs. 15.19; p < 0.001) and therapeutic commitment (24.15 vs. 21.75; p < 0.001). 89 % of the professionals agreed SBI to be useful in PHC.

**Conclusions**: Professionals welcome SBI for drugs but foresee the same implementation barriers as those described when implementing alcohol and tobacco SBI programs. Having previous experience on alcohol and tobacco SBI programs is an advantage when it comes to implement SBI in drugs. Despite limitations due to sampling biases, professionals’ attitudes found, allow some optimism towards a wide implementation of a SBI program on drugs in Catalonia.

## A33 ASSIST feasibility study in primary health care in Catalonia

### Joan Colom-Farran^1^, Nuria Ibañez-Martinez^1^, Juan Manuel Mendive-Arbeloa^2^, Manel Anoro-Perminger^3^, Pako Diaz-Gallego^4^, Lidia Segura-Garcia^1^

#### ^1^Catalonia Department of Health, Public Health Agency, Government of Catalonia, Barcelona, Barcelona, 08005, Spain; ^2^La Mina Primary Health Center, Catalan Institute of Health, Government of Catalonia, Sant Adria de Besos, Barcelona, 08930, Spain; ^3^Besos Primary Health Center, Catalan Institute of Health, Government of Catalonia, Barcelona, Barcelona, 08019, Spain; ^4^Larrard Primary Health Center, PAMEN and Barcelona City Council, Government of Catalonia, Barcelona, Barcelona, 08024, Spain

**Correspondence:** Joan Colom-Farran - joan.colom@gencat.cat

*Addiction Science & Clinical Practice* 2016, **11(Suppl 1)**: A33

**Background**: Among Catalan population aged 15–64 years old, 9 % consume cannabis and 1.6 % cocaine. Despite this, PHC professionals do not screen their patients on their drugs use. The ASSIST screening tool allows the identification of low risk, risk and high risk substance use in non specialized settings. Its validation in Spain showed appropriate sensitivity and specificity levels. This is a feasibility study aimed at exploring the usefulness and acceptability of the ASSIST in the context of PHC of Catalonia prior to its wide-implementation.

**Materials and methods**: It is an observational pilot study with a cross-sectional design. The recruitment of professionals was done through the alcohol referents network. Interested professionals were asked to sign informed consent and invited to a 5-h training covering SBI theory and skills. A web-based ASSIST version was developed and professionals were asked to recruit patients in their normal consultation.

**Results**: 78 out of 112 professionals (67 % nurses and 33 % GP) are actively recruiting. 446 patients have been recruited (55 % male and 44 % female), males aged 41 (SD 15.7) and females 42 (SD 12). 53 % (n = 236) scored positive for risky tobacco consumption, 20 % (n = 90) for alcohol, 19.9 % (n = 89) for cannabis, 7.4 % (n = 33) for non-prescribed sedatives and 6.7 % (n = 30) for cocaine. Risk users received a leaflet with health risk information and recommendations to reduce risks. BI was implemented in 27 % (n = 137) of risk users. Only 0.6 % (n = 3) of the patients refused to receive any type of information.

**Conclusions**: Prevalence rates found are higher than population based ones but they are similar to those found in the ASSIST validation study in Spanish. Despite the sampling bias, PHC professionals see the ASSIST tool as a useful and user-friendly and results obtained facilitate the identification of risk groups to whom the SBI program on drugs should be tailored and targeted.

## A34 **Pilot unit for patients admitted with alcohol intoxication in a tertiary hospital**: **an opportunity to deliver brief intervention**

### Marianthi Deligianni^1^, Nicolas Bertholet^1^, Bertrand Yersin^2^, Jean-Bernard Daeppen^1^, Angeline Adam^1^

#### ^1^Alcohol Treatment Center, Lausanne University Hospital, Lausanne, Vaud, 1011, Switzerland; ^2^Emergency Department, Lausanne University Hospital, Lausanne, Vaud, 1011, Switzerland

**Correspondence:** Marianthi Deligianni - marianthi-lousiana-s.deligianni@chuv.ch

*Addiction Science & Clinical Practice* 2016, **11(Suppl 1)**: A34

**Background**: The aim is to describe patients admitted to a pilot unit of four beds, run by nurses, in a tertiary referral hospital in Switzerland. The unit was open three nights/week (Thursday–Saturday), 22PM–2PM (next day). It was designed to admit medically stable patients with alcohol intoxication for observation, brief intervention delivery and medical evaluation in the morning. Patients could be admitted directly (Glasgow Coma Scale >13) or referred by the Emergency Department (ED) (GCS > 13, modified to GCS > 11 after 3 months).

**Materials and methods**: Between April and December 2015, patient characteristics, ICD-10 alcohol medical diagnoses, brief intervention delivery, and discharge data were recorded.

**Results:** 168 patients were admitted. Mean (SD) age was 33.4 (14.8), 117 (69.6 %) were men. For the 155 for whom alcohol breath testing was possible, the mean breath alcohol concentration was 0.148 % (0.08). Most patients (n = 126, 75.0 %) were referred by the ED, 12 (7.1 %) presented behavioral disorders, with 3 requiring a security guard intervention; 43 (25.6 %) were diagnosed with intoxication with medical complication (F10.01–05), 11 (6.6 %) with harmful use (F10.1) and 35 (20.8 %) with dependence (F10.2); 155 (92.3 %) received a brief intervention; 24 (14.3 %) agreed to an appointment with an addiction specialist. Most were discharged home (n = 145, 86.3 %), 7 (4.2 %) to a shelter/street, 6 (3.6 %) were transferred to the ED, 4 (2.4 %) to a psychiatric inpatient unit, and 6 (3.6 %) to a psychiatry outpatient unit.

**Conclusion**: Most patients received a brief intervention as intended. Most patients did not require intensive medical care and were discharged home. This unit can contribute in avoiding unnecessary admissions to the ED, while offering an opportunity to link patients with preventive and specialized addiction care, even though the acceptance rate of addiction specialist referral was limited.

## A35 The role of primary care screening in ongoing care management of alcohol and drug problems

### Constance Weisner^1,2^, Felicia Chi^2^, Wendy Lu^2^, Stacy Sterling^2^

#### ^1^Department of Psychiatry, University of California, San Francisco, CA, 94143, USA; ^2^Division of Research, Kaiser Permanente Northern California, Oakland, CA, 94612 USA

**Correspondence:** Constance Weisner - Constance.Weisner@KP.org

*Addiction Science & Clinical Practice* 2016, **11(Suppl 1)**: A35

**Background**: Objectives of SBIRT include identifying individuals with alcohol and drug (AOD) problems and facilitating specialty treatment. However, screening in primary care may play an important role in ongoing AOD care as well.

**Materials and methods**: We conducted a trial of 503 adults in specialty AOD treatment in a large Northern California health system. The LINKAGE arm substituted the program’s six group-based usual care (UC) medical education sessions with six group-based sessions based on patient activation and use of health-information technology resources in the electronic health record (EHR). LINKAGE participants were also offered a facilitated encrypted email or telephone visit with their physician. The primary outcome was engagement in health care. We also examined communication with the patients’ primary care physicians about their AOD use.

**Results**: Patients in the LINKAGE condition reported more communication with their physicians about AOD use (70.5 vs. 51 %, p < .001) at 6 months. Results from GEE overdispersed Poisson models found, compared to UC, LINKAGE participants showed an 1.53 fold increase in average patient portal log-in days (IRR = 1.53, 95 % CI: 1.19, 1.97, p = 0.0014) similar findings were found across types of EHR patient portal use: e.g., physician email (IRR = 1.45, 95 % CI: 1.08, 1.94, p = 0.017). Results were similar at 12 months, e.g., 47.85 versus 37.11 %, p = .03 for physician communication about AODs, and 83 versus 70 %, p < .001 for patient portal use). Preliminary results at 24 months are similar. Findings across time points were similar for sub-groups with chronic medical and psychiatric conditions, and by gender.

**Conclusions**: For those in specialty AOD treatment, patient activation approaches and providing facilitation in linking with health care may result in ongoing communication with primary care physicians, thus motivating ongoing screening and intervening in primary care. This may be an important complement to training primary care physicians. The role of patients is important in ongoing care.

*Trial registration*: clinicaltrials.gov Identifier:NCT01621711

## A36 Initiation, engagement, and retention in substance use disorder treatment in HIV infected and uninfected patients

### Kevin L. Kraemer^1^, Kathleen A. McGinnis^2^, David A. Fiellin^2^, Melissa Skanderson^2^, Adam J. Gordon^1,3^, Jonathan Robbins^1^, Susan Zickmund^1,3^, P. Todd Korthuis^4^

#### ^1^Department of Medicine, University of Pittsburgh, Pittsburgh, PA, 15213, USA; ^2^Department of Medicine, Yale University, New Haven, CT, 06520, USA; ^3^Center for Health Equity Research and Promotion, VA Pittsburgh Healthcare System, Pittsburgh, PA, 15261, USA; ^4^Department of Medicine, Oregon Health Sciences University, Portland, OR, 97239, USA

**Correspondence:** Kevin L. Kraemer - kraemerkl@upmc.edu

*Addiction Science & Clinical Practice* 2016, **11(Suppl 1)**: A36

**Background**: Substance use disorders (SUD) are common among HIV-infected and uninfected patients, but are often untreated. To assess differences in receipt of care, we compared the frequency of initiation, engagement, and retention in SUD treatment between HIV-infected and uninfected patients.

**Materials and methods**: We used electronic national Veterans Administration (VA) data (years 2000–2012) from 43,116 HIV-infected and 94,253 uninfected (matched 1:2) participants in the Veterans Aging Cohort Study. We defined new SUD episodes as outpatient visits or inpatient/residential admissions with associated primary or secondary substance use ICD-9 codes following a break in SUD care (defined as 5 months without a SUD-related service or pharmacotherapy). We used established performance indicators to define initiation, engagement, and retention in SUD treatment. We used descriptive and bivariate statistics to compare initiation, engagement, and retention between HIV-infected and uninfected.

**Results**: The sample was primarily male (97 %) and racially diverse (39 % white, 48 % black, 7 % Hispanic). A greater proportion of HIV-infected than uninfected had at least one new SUD episode (37.6 vs. 34.4 %; p < .001). Following a new SUD episode, HIV-infected were slightly more likely to initiate (16.2 vs. 15.8 %; p < .05), engage (19.9 vs. 18.9 %; p < .01), and retain (20.9 vs. 19.7 % at 3 months; 17.4 vs. 16.4 % at 6 months; p < .01 for both) in SUD treatment than uninfected. HIV-infected participants were less likely than uninfected to initiate any SUD-specific pharmacotherapy (1.6 vs. 2.3 %; p < .001).

**Conclusions**: In this national VA sample, the rates of initiation, engagement, and retention in SUD treatment were low for both HIV-infected and uninfected. Although we could not assess the SBI component of SBIRT in this dataset, our findings suggest the “referral-to-treatment” (RT) component is sub-optimal for both patient groups. Interventions to improve referral-to-treatment and access to SUD treatment are needed.

## A37 Integrated stepped care for unhealthy alcohol use in HIV clinics

### E. Jennifer Edelman^1,2^, Nathan B. Hansen^2,3^, Christopher J. Cutter^1^, James Dziura^4^, Lynn E. Fiellin^1,2^, Patrick G. O’Connor^1^, Stephen A. Maisto^5^, Roger Bedimo^6^, Cynthia Gilbert^7^, Vincent C. Marconi^8^, David Rimland^8^, Maria Rodriguez-Barradas^9^, Michael Simberkoff^10^, Amy C. Justice^1,11^, Kendall J. Bryant^12^, David A. Fiellin^1,2^

#### ^1^Yale University School of Medicine, New Haven, CT, 06510, USA; ^2^Center for Interdisciplinary Research on AIDS, Yale University School of Public Health, New Haven, CT, 06510, USA; ^3^College of Public Health, University of Georgia, Athens, GA, 30602, USA; ^4^Yale Center for Analytic Sciences, Yale University School of Public Health, New Haven, CT, 06511, USA; ^5^Syracuse University, Syracuse, NY, 13244, USA; ^6^Veterans Affairs North Texas Health Care System and UT Southwestern, Dallas, Texas, 75216, USA; ^7^D.C. VAMC and George Washington University, Washington, District of Columbia, 20422, USA; ^8^Atlanta VAMC and Emory University School of Medicine, Atlanta, Georgia, 30033, USA; ^9^Michael E. DeBakey VAMC and Baylor College of Medicine, Houston, Texas, 75216, USA; ^10^VA NY Harbor Healthcare System and New York University, New York, NY, 10010, USA; ^11^VA Connecticut Healthcare System, Veterans Aging Cohort Study, West Haven, CT, 06516, USA; ^12^National Institute on Alcohol Abuse and Alcoholism HIV/AIDS Program, Bethesda, MD 20892-7003, USA

**Correspondence:** E. Jennifer Edelman - ejennifer.edelman@yale.edu

*Addiction Science & Clinical Practice* 2016, **11(Suppl 1)**: A37

**Background**: Effective alcohol treatment interventions are rarely offered in HIV treatment settings, yet are needed.

**Materials and methods**: We are conducting three linked randomized controlled trials in five Veteran Affairs-based Infectious Disease Clinics. Based on AUDIT-C and Timeline Followback data, participants meeting criteria for: (1) at-risk drinking, (2) moderate alcohol use with liver disease (MALD) (>1 drinks in past 30 days, evidence of liver fibrosis with a FIB-4 score >1.45 or Hepatitis C-coinfection), or (3) alcohol use disorder (AUD) are randomized to integrated stepped care (ISC) versus treatment as usual. For those with at-risk drinking or MALD, ISC starts with a Brief Intervention and 2-week telephone booster. Based on pre-specified nonresponse criteria, participants may be “stepped up” at week 4 to receive four sessions of motivational enhancement therapy (MET) and may be “stepped up” again at week 12 for Addiction Physician Management (APM) and alcohol pharmacotherapy. For those with AUD, ISC begins with APM. Non-responders may be “stepped up” at week 4 to receive MET and again at week 12 for a higher level of care (e.g. intensive outpatient program). Primary outcome is alcohol consumption, secondary outcome is VACS Index, a validated measure of HIV morbidity and mortality risk. We used descriptive statistics to characterize enrolled participants at baseline.

**Results**: Participants (N = 280) are a mean age of 58 years, and predominantly black (82 %), non-Hispanic (93 %) men (98 %). At baseline, those with at-risk drinking (n = 77) had a mean of 20 drinks/week, those with MALD (n = 89) had a mean of 4 drinks/week and those with AUD (n = 113) had a mean of 31 drinks/week (p < 0.001). The proportion with a detectable HIV viral load was 31, 23 and 36 %, respectively (p = 0.098).

**Conclusions**: If effectiveness is demonstrated, future efforts should focus on implementation of this novel approach to addressing unhealthy alcohol use in HIV clinics.

*Trial registration*: NCT01410123

## A38 **Using the AUDIT, AUDIT-C and TLFB as outcome measures in intervention trials**: **A discussion of current challenges and benefits**

### Anne H. Berman^1^, Gillian W. Shorter^2^

#### ^1^Karolinska Institutet, Department of Clinical Neuroscience, Center for Psychiatry Research, 113 64 Sweden; ^2^Trinity College Dublin, University of Dublin, Dublin 2, Ireland

**Correspondence:** Anne H Berman - anne.h.berman@ki.se

*Addiction Science & Clinical Practice* 2016, **11(Suppl 1)**: A38

**Background**: To understand if our interventions are effective, we need alcohol outcome measures that are valid, reliable, psychometrically sound, and fit for purpose.

**Materials and methods**: This presentation will discuss three commonly used measures AUDIT, AUDIT-C, and Timeline Followback (TLFB) to review suitability for mapping meaningful alcohol change in brief intervention trials.

**Results**: The 10-item AUDIT and 3-item AUDIT-C provide a useful summary of different aspects of alcohol consumption, including negative consequences in the AUDIT questionnaire. Psychometric properties, particularly for the full AUDIT, are very good for estimating alcohol use and related problems in the past year. In research, the AUDIT is useful for baseline measures but in studies with follow-ups at three or 6 months, psychometric properties could be compromised by adapting the questions to fit. The TLFB can be used repeatedly as a measure of consumption over the past 7-days, or extended to fit differing time frame measures. There are differences in respondent burden; in AUDIT or AUDIT-C participants may have to average their consumption where there is no typical drinking occasion. For TLFB, the researcher calculates total drinks in a given time period, but the time taken for the respondent to recall drinking and complete the questionnaire is longer. Additionally all three instruments are based on self-report; objective evidence from biomarkers might be a helpful complement to validate findings.

**Conclusions**: Several issues will be discussed, including the merits of adapting instruments to better measure alcohol-related change in intervention trials, and how this might be achieved. There is also a gap regarding overlap between the different instruments, and how this affects comparability of outcome measures given that researchers choose different measures. The talk will reflect on the extent to which current use of these measures limits our understanding and ability to compare effectiveness across trials of different interventions.

## A39 **Non-alcohol outcomes**: **What are the most relevant in the context of alcohol brief intervention trials?**

### Aisha Holloway

#### School of Health in Social Science, The University of Edinburgh, Edinburgh, EH8 9AG, Scotland, UK

**Correspondence:** aisha.holloway@ed.ac.uk

*Addiction Science & Clinical Practice* 2016, **11(Suppl 1)**: A39

**Background**: The importance of non-health outcomes and specifically non-alcohol outcomes is gaining increasing attention, with the latter in relation to the effect measures of alcohol brief interventions. Non-alcohol outcomes are not commonly employed as outcome measures in economic evaluations, as is the case with generic instruments such as Quality Adjusted Life Years (QALYs), EQ-5D or SF-36. There is also a lack of literature identifying the most relevant non-alcohol outcomes for alcohol brief interventions.

**Materials and methods**: This presentation explores and considers possible non-alcohol outcomes for alcohol brief intervention trials by drawing from existing literature on non-health outcomes.

**Results**: Non-health outcome measures identified include: self-confidence; insights into own un(healthy) behavior; perceived life control; knowledge about a certain health problem; social support; relaxation; better educational achievements; increased labor participation and work productivity; social participation and a reduction in criminal behavior. These are also seen elsewhere in the literature with outcome descriptions worded differently. It is also reported in the literature that, when considering such outcomes, it is necessary to be cognizant of the particular subgroups of individuals and populations that the specific intervention is for. This is particularly relevant for alcohol brief interventions where settings for implementation range from primary care to criminal justice, workplace, schools and general hospital settings, with sub-populations varying in relation to gender, age range and ethnicity. Likewise, the relevance of the outcomes to the individual, direct social environment, and society as a whole, are also important considerations.

**Conclusions**: This presentation draws from the non-health outcome literature to provide insights into the type of non-alcohol outcome measures that could be relevant in the context of alcohol brief interventions, mindful of the need for highly reliable and valid instruments that measure such outcomes.

## A40 Measuring economic outcomes in alcohol screening and brief intervention studies

### Jeremy W. Bray^1^, Carolina Barbosa^2^

#### ^1^The University of North Carolina at Greensboro, Greensboro, NC 27412, USA; ^2^RTI International, Chicago, IL 60606, USA

**Correspondence:** Jeremy W Bray - jwbray@uncg.edu

*Addiction Science & Clinical Practice* 2016, **11(Suppl 1)**: A40

**Background**: There is a widespread belief that alcohol screening and brief intervention (ASBI) is both cost-effective and cost-beneficial. Yet the evidence supporting these conclusions is weak. Recent reviews suggest that cost-effectiveness evidence for ASBI in emergency care and hospital settings is scarce and that it is unclear whether ASBI for alcohol misuse will result in net cost savings. These reviews are hampered by the lack of consistent measures of economic outcomes across studies. In this presentation, we review the outcomes used in previous ASBI economic evaluations and discuss the role of these measures in the broader INEBRIA Research Measurement Standardization Group (IRMSG) effort.

**Materials and methods**: We reviewed two related literatures to assess the state of economic outcome measurement in ASBI studies. The first consists of ASBI economic evaluations, which we reviewed to identify economic outcome measures used in the ASBI field. The second consists of studies reporting the social costs of alcohol use and use disorders, which we reviewed to determine the economic outcomes most associated with the social costs of alcohol misuse.

**Results**: We find that ASBI economic evaluations seldom use consistent measures, but an increasing number of studies report quality adjusted life years (QALYs) in addition to measures of social costs. Social costs studies suggest that the IRMSG should consider measures of: health state utility as derived from health-related quality of life; health care use; injuries and accidents, including motor vehicle accidents; crime and criminal justice involvement; employment, workplace productivity, and absenteeism; and, for adolescent studies, educational outcomes such as school attendance and matriculation.

**Conclusions**: To support the development of a rigorous evidence base for the economic benefits of ASBI, the IRMSG should develop a core set of economic outcome measures linked to social cost studies.

## A41 The effects of extended internet based interventions designed to reduce alcohol problems – a systematic review

### Magnus Johansson

#### Department of Medicine, Karolinska Institutet, Stockholm, 17177, Sweden

**Correspondence:** magnus.johansson.1@ki.se

*Addiction Science & Clinical Practice* 2016, **11(Suppl 1)**: A41

**Background:** Internet-based interventions for problematic alcohol use have been studied for a number of years. Internet-based brief interventions (eSBIs), the most studied format, are usually one single session and mainly focused on screening of alcohol use and personalized normative feedback to the user. eSBIs have been shown to be effective in reducing alcohol consumption, at least in the short term. More extended interventions intended to be used continually for a number of weeks or months to decrease alcohol consumption have reported slightly larger effects but been less studied.

**Materials and methods:** A systematic review was conducted late 2015. A wide search strategy was used in PubMed, PscyhInfo, WebofScience and Cochrane. Randomized controlled trials and observational studies using extended internet- or computer based interventions were included.

A total of 612 articles were identified and their abstracts coded, 55 articles were read in full text. Population characteristics, interventions, retention and attrition, non-responders or deterioration, use of other support, effects and study quality were extracted into a table and summarized and quality of studies assessed.

**Results:** 53 articles reporting 39 trials of 32 interventions where included in this review. An additional 20 studies in primary care settings will be reviewed separately. The included studies involved 21,496 participants and were all conducted in Western, high income countries. Participants had diagnosed alcohol use disorder or AUDIT-score corresponding with harmful use or dependence. Most of the interventions based on MI/CBT. 27 were randomized controlled trials.

**Conclusions:** Extended computer or internet based interventions are associated with reduced drinking, increased abstinence and reduced alcohol-related problems. But the evidence on this association being causal is not conclusive and several other factors that could explain the effects have to be further investigated.

## A42 **Digital approaches in primary care**: **early findings from the implementation of www.checkupandchoices.com web app**

### Reid Hester, William Campbell

#### Research Division, Checkup & Choices, LLC, Albuquerque, NM, 87112, USA

**Correspondence:** Reid Hester - reid@checkupandchoices.com

*Addiction Science & Clinical Practice* 2016, **11(Suppl 1)**: A42

**Background**: Healthcare organizations are interested in providing digital interventions to patients with alcohol problems once they have screened positive for problem drinking in primary care settings. Digital interventions are seen as the first step in a stepped care model of treatment (Sobell & Sobell 2000). They can also be used as adjuncts to face-to-face interventions in the SBI model. Digital interventions have the potential to provide clinically effective interventions for problem drinkers with few additional demands on clinic resources.

**Methods**: We are beginning implementation pilot programs in two healthcare organizations in New Mexico and California (April, 2016). We are tailoring how our empirically supported web app www.checkupandchoices.com will be provided to clinic patients and will be learning the needs of providers for clinical feedback. This dissemination and implementation will be an iterative process undertaken with the healthcare organizations and healthcare providers as collaborators. And each pilot will be unique in its structure. We plan to document the implementation process as it unfolds using the Comprehensive Framework for Implementation Research (CFIR; http://www.cfirguide.org).

**Results**: While we are in the beginning stages, we have already learned that: (a) developing the implementation plans takes months, involves numerous stakeholders, and has involved every division of our company; (b) customizing the app takes time and IT resources; c) how the app will be integrated into the workflow of each system is unique.

**Conclusions**: The outcome data will demonstrate the feasibility (or lack thereof) of implementing the www.checkupandchoices.com web app in these two primary care settings. The lessons learned will help us better address the challenges in implementation in future projects. The lessons learned can also provide a framework that could be used both in other healthcare settings and with other digital tools for screening and brief intervention.

## A43 Digital approaches for people with alcohol problems

### Maria Lucia O. Souza Formigoni^1^, André Luzi Monezi Andrade^1^, Laisa Marcorela Andreoli Sartes^2^

#### ^1^Departamento de Psicobiologia - Universidade Federal de São Paulo, São Paulo, 04021-001 Brazil; ^2^Departamento de Psicologia - Universidade Federal de Juiz de Fora, Juiz de Fora, 36036-900 Brazil

**Correspondence:** Maria Lucia O. Souza Formigoni - mlformig@gmail.com

*Addiction Science & Clinical Practice* 2016, **11(Suppl 1)**: A43

**Background**: The use of digital approaches in the screening of substance use, and to deliver brief interventions, has increased in recent years, due to its relatively low cost and potential to deal with outreach people. The increased use of Internet allows delivering intervention both to people in isolated places or to those who do not look for health services due to fear of stigmatization. There is some evidence of efficacy of virtual interventions to tobacco, alcohol, cannabis and cocaine users, despite a high dropout rate, but a paucity of data on the profile of the users who adhere to these interventions, and a limited number of randomized controlled trials on their effectiveness.

**Materials and methods**: We developed a pilot study in Brazil on the adherence to and effectiveness of a virtual intervention (www.bebermenos.org.br, meaning drinking less in Portuguese) as part of a multicenter international project developed in Brazil, Mexico, India and Belarus, supported by the World Health Organization. We collected data on the acceptability of the site by Brazilian users from 2013 to 2015. The participants fulfilled the AUDIT screening instrument and during 6 weeks had access to tools designed to monitor their consumption, detect risk situations and develop strategies to avoid them and reduce alcohol ingestion.

**Results**: Out of 41,147 people who accessed the site, 4148 signed into start the virtual intervention and 1327 agreed to participate in the study. Out of these, 22 % fulfilled the follow-up form. Those classified as harmful users reduced their alcohol consumption in 36 % when compared to their baseline levels, while those classified as suggestive of dependence reduced it to 48 %.

**Conclusions**: High baseline consumption was associated with success. New strategies must be developed to increase the adherence to this kind of intervention.

## A44 Guided and unguided internet-based cognitive behavioral therapy for problematic alcohol use

### Christopher Sundström^1^, Niels Eék^2^, Martin Kraepelien^1^, Viktor Kaldo^1^, Claudia Fahlke^2^, Anne H. Berman^1^

#### ^1^Department of Clinical Neuroscience, Karolinska Institutet, 171 77 Solna, Sweden; ^2^Department of Psychology, Gothenburg University, 405 30 Gothenburg, Sweden

**Correspondence:** Anne H Berman - anne.h.berman@ki.se

*Addiction Science & Clinical Practice* 2016, **11(Suppl 1)**: A44

**Background**: For several years, the Internet has been studied as mode of delivery for interventions of different length and intensity targeting problematic alcohol use. Most interventions studied have been single-session and fully automated, but research suggests that multi-session programs are more beneficial than single sessions, and that adding therapist guidance to the intervention improves alcohol consumption outcomes.

**Materials and methods**: In a pilot study conducted in the spring of 2015, an extensive 12-module internet-based program (ePlus) based on cognitive behavioral therapy (CBT) and relapse prevention (RP) was tested among Internet help-seekers. Thirteen participants, recruited online from an open access website and fulfilling at least two DSM-5 diagnostic criteria for Alcohol use disorder (AUD), were all offered the ePlus program in combination with online therapist support by licensed psychologists. Since January 2016, a randomized controlled trial (RCT) is being conducted where 169 participants fulfilling AUD diagnostic criteria and recruited from ads on the internet are randomized into three groups: a ‘high intensity’ intervention (ePlus with therapist support as in the above-mentioned pilot trial), a ‘low intensity’ intervention (eChange; a briefer CBT/RP program without therapist support) or to a wait list control group.

**Results**: Preliminary results at the 6-month post-randomization follow-up indicate that the program was beneficial, as mean alcohol consumption dropped from 23.1 glasses per week to 5.1 glasses per week and AUDIT scores dropped from 23.7 to 10.9. Furthermore, post-treatment interviews and questionnaires displayed high acceptability among users. Regarding the RCT, 70 participants were recruited to the study during its first 4 months, recruitment will be completed in the spring of 2017. Follow-up will be conducted immediately post-treatment and at 3, 6, 12 and 24 months post-treatment.

**Conclusions**: The results from these studies will add to knowledge regarding the effectiveness of high- versus low-intensity interventions, with or without guidance, for reducing problematic alcohol consumption fulfilling AUD criteria.

*Trial registration*: Clinicaltrials.gov NCT02645721

## A45 Moderators of brief motivation-enhancing treatments for alcohol-positive adolescents presenting to the emergency department

### Lynn Hernandez^1,2^, Sara J. Becker^1^, Richard N. Jones^2^, Hannah R. Graves^2^, Anthony Spirito^1,2^

#### ^1^Center for Alcohol and Addiction Studies, Brown University, Providence, RI, 02912, USA; ^2^Department of Psychiatry and Human Behavior, Brown University, Providence, RI, 02912, USA

**Correspondence:** Sara J. Becker - sara_becker@brown.edu

*Addiction Science & Clinical Practice* 2016, **11(Suppl 1)**: A45

**Background**: The emergency department (ED) visit offers a unique opportunity to screen adolescents for alcohol problems and offer brief intervention. A 2011 randomized controlled trial compared the effectiveness of two brief motivation enhancing therapy (MET) models among alcohol-positive adolescents in an urban ED: adolescent MET-only versus MET+ Family Check Up (FCU), a parent MET model. Results indicated that among the 97 adolescents completing the 3-month assessment, both conditions were associated with reduced drinking and MET + FCU was associated with lower rates of high volume drinking than adolescent MET-only. The goal of this study was to identify predictors and moderators of high volume drinking in the original trial.

**Methods**: Adolescents were recruited in an urban level I trauma center in the Northeast United States. To be eligible, adolescents needed to report drinking alcohol in the 6 h before the ED visit or exhibit a positive blood alcohol concentration as tested using blood, breath, or saliva. One hundred and thirteen adolescents (90 % of randomized) received the allocated intervention and 97 (78 % of randomized, 86 % of treated) completed the 3-month assessment and were analyzed in the original trial.

**Results**: Seven candidate variables were evaluated as moderators across three domains: demographic characteristics, psychological factors, and socio-contextual factors. Analyses of covariance models identified one significant predictor and one significant moderator of outcome. Older adolescents had significantly worse drinking outcomes than younger adolescents regardless of MET condition. Adolescents whose parents screened positive for problematic alcohol use at baseline had significantly worse drinking outcomes in the MET + FCU condition than the MET-only condition.

**Conclusions**: Results indicate that alcohol-positive adolescents presenting to the ED may respond better to MET models if they are under the age of 16. Involving parents who have problematic alcohol use in a parent-focused MET may have negative effects on adolescent high volume drinking.

## A46 Moderators of effectiveness in a brief motivational intervention for alcohol intoxicated adolescent ED patients

### Silke Diestelkamp, Lutz Wartberg, Nicolas Arnaud, Rainer Thomasius

#### German Center for Addiction Research in Childhood and Adolescence, University Clinic Hamburg Eppendorf, Hamburg, D-20246, Germany

**Correspondence:** Silke Diestelkamp - s.diestelkamp@uke.de

*Addiction Science & Clinical Practice* 2016, **11(Suppl 1)**: A46

**Background**: Rising numbers of adolescents receiving emergency medical treatment due to acute alcohol intoxication have been a major public health concern in a range of European countries in recent years. Brief interventions (BIs) addressing this target population have been introduced in a number of emergency departments with the aim to reduce alcohol-related harm. However, evidence of effectiveness of this approach is inconclusive. Moderator analyses are a means to shed light on variables influencing effectiveness. Therefore, this study examined potential moderators of BI effectiveness and hereby addressed client variables, counselor variables and intervention content.

**Materials and methods**: Data from the randomized controlled “HaLT-Hamburg” trial which evaluated a manualized brief motivational intervention for adolescents following alcohol intoxication in the emergency department were analyzed. As potential moderators client variables (age, gender, habitual drinking, psychosocial problems), counselor variables (empathy, affirmation, competence, congruence), and intervention content (use of the confidence and readiness ruler, decisional balance, goal setting) were tested. Moderation analyses were conducted applying multiple regression analysis for counselor variables and intervention content. Client variables were examined applying mixed-effects analysis of covariance (ANCOVA) models adjusted for the respective client variables.

**Results**: Data of N = 316 adolescents (Mean age 15.8 (SD = 1.2), 52 % male) were examined. Age, gender, habitual drinking and psychosocial problems did not moderate intervention effectiveness. Examination of counselor variables and intervention content revealed that higher scores on the basic therapeutic skill “positive affirmation” and finishing the intervention with a written goal setting agreement were associated with greater readiness to change alcohol use.

**Conclusions**: Results support prior findings on the influence of counselors’ adherence to MI spirit, such as “affirmation”, on clients’ motivation to change. Further research into variables influencing BI effectiveness in the target group of underage drinkers addressed in the emergency department is needed.

*Trial registration*: Current Controlled Trials ISRCTN31234060

## A47 A review of active ingredients and a qualitative design to develop a brief alcohol intervention for young adults intoxicated in the ED

### Jacques Gaume, Sophie Paroz, Véronique Grazioli, Cristiana Fortini, Nicolas Bertholet, Jean-Bernard Daeppen

#### Alcohol Treatment Center, Department of Community Health and Medicine, Lausanne University Hospital, Lausanne, 1011, Switzerland

**Correspondence:** Jacques Gaume - jacques.gaume@chuv.ch

*Addiction Science & Clinical Practice* 2016, **11(Suppl 1)**: A47

**Background**: Harmful alcohol use among young adults is a major public health concern and accounts for a significant portion of disease burden in Switzerland and worldwide. In Switzerland, Emergency Department (ED) admissions for alcohol intoxication have increased substantially over the past decade, particularly among adolescents and young adults. Brief motivational interventions (BMI) for young adults conducted in the ED have shown promising but inconsistent results.

**Materials and methods**: We reviewed the literature on brief intervention and motivational interviewing active ingredients in order to better understand which ingredients may be related to better outcomes. Based on this knowledge base, we developed a new motivational intervention model for young adults admitted in the ED with alcohol intoxication. Using a qualitative design, we pre-tested this model by conducting 5 experimental sessions to evaluate the interventionists’ and patients’ experience.

**Results**: Overall, BMI active ingredients have been scarcely investigated among young ED patients. Based on this literature and broader behavioral interventions’ mechanisms research, we found 6 axes for a new model development: 1. High level of relational factors (e.g. empathy, alliance, collaboration; avoidance of confrontation); 2. Personalized feedback (only if patient is willing to receive it); 3. Enhance discrepancy; 4. Evoke change talk while softening sustain talk, strengthen ability and commitment to change; 5. Completion of a change plan (only if patient is ready for change); 6. Devote more time: longer sessions, follow-up options (face-to-face, telephone, or electronic boosters; referral to treatment). Qualitative analysis of experimental sessions gave important insights regarding acceptability and feasibility of the model.

**Conclusions**: The review provided valuable insights on which active ingredients may be related to better outcomes. Based on it, we proposed an updated model to be further tested. Future plans include an experts’ consultation on the model ingredients, additional experimental sessions, and a full randomized controlled trial testing our updated model.

## A48 Development, implementation and evaluation of a training intervention for primary care providers on brief behaviour change counselling, and assessment of the provider’s competency in delivering this counselling intervention

### Zelra Malan^1^, Bob Mash^1^, Katherine Everett-Murphy^2^

#### ^1^Division of Family Medicine and Primary Care, Stellenbosch University, Stellenbosch, 8000, South Africa; ^2^Chronic Diseases Initiative in Africa (CDIA), Faculty of Health Sciences, Cape Town University, Cape Town, 7701, South Africa

**Correspondence:** Zelra Malan - zmalan@sun.ac.za

*Addiction Science & Clinical Practice* 2016, **11(Suppl 1)**: A48

**Background:** Unhealthy behavior is a key modifiable factor that underlies much of the global burden of disease and primary care morbidity. Chronic diseases (NCDs) are linked to underlying behavioral issues such as tobacco smoking, alcohol abuse, physical inactivity and unhealthy eating.

Evidence shows that brief behavior change counseling (BBCC) by primary care providers (PCP) can be effective in helping patients to change risky lifestyle behaviors. However, the capacity of South African PCP’s to educate and counsel patients on lifestyle modification is generally poor.

The aim of this research was to analyze the current situation, design, develop, implement and evaluate the effectiveness of a training intervention for PCPs to offer patients BBCC on the lifestyle risk factors associated with NCDs.

**Materials and methods:** The ADDIE model provided a conceptual model for the research. The steps were: analysis of learning needs, the design and development of the training program, and finally the implementation and evaluation of PCPs counseling performance after training in clinical practice.

**Results:** A best practice BBCC training program was designed, developed and implemented in our context, targeting either clinical nurse practitioners or primary care doctors. The training combined the 5As (ask, alert, assess, assist and arrange) with the guiding style of motivational interviewing. The training was effective at changing PCPs clinical practice, in the short term. Training also changed PCPs perception of their ability to offer BBCC, and increased their confidence to overcome certain barriers to implementation, for instance time constraints.

**Conclusions:** Training alone is not enough to ensure implementation of BBCC at the coal face. Successful implementation of BBCC not only requires training PCPs to change their counseling behaviors, but also requires change at other levels. To incorporate BBCC into everyday care a whole systems approach is needed, specifically changing the underlying supportive culture in primary care settings.

## A49 **The interactive effects of personality traits and peer influence on alcohol use**: **Which young men might benefit most from interventions targeting peer-influence?**

### Véronique S. Grazioli^1^, Joseph Studer^1^, Meichun Mohler-Kuo^2^, Jean-Bernard Daeppen^1^, Nicolas Bertholet^1^, Gerhard Gmel^1^

#### ^1^Department of Community Medicine and Health, Lausanne University Hospital, Lausanne, 1011, Switzerland; ^2^Institute of Social and Preventive Medicine, and Epidemiology, Biostatistics and Preventive Institute, University of Zurich, Zurich, 8001, Switzerland

**Correspondence:** Véronique S. Grazioli - Veronique.Grazioli@chuv.ch

*Addiction Science & Clinical Practice* 2016, **11(Suppl 1)**: A49

**Background**: Peer influence (PI) is a factor that can be targeted in brief interventions. PI is a robust predictor of drinking, yet not all young adults are exposed to its influence in the same way. This study aimed at identifying young adults who are more vulnerable to PI by prospectively examining whether personality traits moderate the associations between PI and alcohol use among young male drinkers.

**Materials and methods**: Participants (N = 4665) were young Swiss male drinkers participating in a larger study—the Cohort Study on Substance Use Risk Factors (C-SURF). Measures of PI—descriptive norms (DNs) and peer pressure to misconduct (PPM), sensation seeking, aggression and alcohol use (total drinks per week) were assessed at baseline and 15 months later.

**Results**: Generalized linear modeling indicated that sensation seeking significantly moderated the associations between (a) DNs and current (b = −0.21, SE = 0.04, p < .001) and future (b = −0.13, SE = 0.04, p < .01) alcohol use, (b) PPM and current alcohol use (b = −0.08, SE = 0.04, p < .05). Aggression significantly moderated the associations between (a) DNs and current alcohol use (b = −0.65, SE = 0.16, p < .001), (b) PPM and current (b = −0.52, SE = 0.16, p < .01) and future alcohol use (b = −0.42, SE = 0.18, p < .05). Although participants scoring higher on the moderators consistently reported higher levels of alcohol use than those scoring lower on these measures, moderation analyses revealed that the associations between PI and alcohol use were generally stronger among participants scoring lower on personality traits.

**Conclusions**: These findings suggest that young male drinkers with low scores on sensation seeking and aggression may benefit from stand-alone selective interventions targeting PI, whereas those scoring higher on these personality traits may rather benefit from programs that both include interventions targeting PI and personality risk factors of alcohol-related behaviors.

## A50 Midwives’ perceptions and attitudes to prenatal alcohol consumption and their influences on screening and brief interventions

### Lawrence Doi^1^, Helen Cheyne^2^, Ruth Jepson^1^

#### ^1^Scottish Collaboration for Public Health Research and Policy, Usher Institute of Population Health Science and Informatics, University of Edinburgh, Edinburgh, EH8 9AG, UK; ^2^Nursing, Midwifery and Allied Health Professionals Research Unit, University of Stirling, Stirling, FK9 4LA, UK

**Correspondence:** Lawrence Doi - larry.doi@ed.ac.uk

*Addiction Science & Clinical Practice* 2016, **11(Suppl 1)**: A50

**Background**: There are mixed messages about the consequences of prenatal alcohol consumption. The Scottish Government implemented screening and brief interventions in antenatal care setting to help address this and reduce alcohol exposed pregnancies. As little is known about how the complexity of conflicting messages influence midwives perceptions and attitudes, this study examined how these factors affect midwives’ delivery of screening and brief interventions.

**Materials and methods**: In this qualitative study, 21 midwives participated in individual interviews and focus groups. Data were analyzed using a hybrid approach of inductive and deductive coding and theme development.

**Results**: Midwives showed a clear understanding of the effects of drinking in pregnancy and were optimistic about their involvement in the screening and brief intervention program. However, they reported that many women had already given up drinking or were drinking minimal amounts prior to their first antenatal appointment. They felt that delivering them pre-pregnancy would be more beneficial. This partially negated the purpose of the program as some midwives accorded screening and brief interventions low priority in their workload, especially in the midst of competing priorities. For women who were currently drinking, midwives indicated that sufficient rapport was necessary at the first antenatal appointment in order to discuss alcohol issues appropriately, but they felt this was often challenging.

**Conclusions**: Although midwives identified several challenges of delivering screening and brief interventions, antenatal setting provides a unique opportunity for screening and delivering brief interventions to address some of the misconceptions of prenatal alcohol consumption, and hopefully reduce drinking in pregnancy. However, several factors may need to be considered to improve their delivery in this setting.

## A51 Efficacy of group SBI for alcohol use disorders versus individual SBI

### Vanesa Luna, Leticia Echeverria, Silvia Morales

#### School of Psychology, National University of Mexico, Mexico City, 04510, Mexico

**Correspondence:** Vanesa Luna - angelvana2004@hotmail.com

*Addiction Science & Clinical Practice* 2016, **11(Suppl 1)**: A51

**Background**: Most of the evaluation into SBI for alcohol problems, at least in Mexico are delivered individually, to one person at a time - the group therapy in many places are actually the most commonly used type of intervention. But in our country we have few studies that can show us the evidence on the effectiveness of group treatment.

The aim of this study is to demonstrate that the group intervention is not only effective but might also less expensive that individual treatment.

We used a cognitive-behavioral approach, that involve learning and coping skills which help people manage triggers that lead them to drink, to prevent relapse and maintain abstinence or low risk drinking.

**Materials and methods**: The sample was integrated by 50 users of alcohol consumers with an average age of 22 years, who came voluntarily to receive the SBI in Addictions Care Center, Faculty of Psychology at the National University of Mexico. 3 groups of SBI was perform.

First it conducted an evaluation session that in both cases was carried out individually, where the quantity and frequency of consumption was evaluated and the consequences of the consumption.

Users then received 4 sessions of cognitive behavioral SBI raises both individual format as the group. Upon completion of the treatment pattern of consumption and the negative consequences reported were evaluated during a follow up after 6 months.

**Results**: The study found no difference in effectiveness between group and individual therapy but group SBI had better cost effectiveness option compare with individual therapy.

**Conclusions**: Group brief interventions is considered a therapy with better cost-effectiveness option compared with individual treatment.

## A52 Types of alcohol-related accidents and episodes of violence in the emergency department of the Central Hospital of São Tomé and Príncipe

### Teresa Barroso^1^, Ângela Abreu^2^, Cosma Aguiar^3^

#### ^1^Mental Health Nursing Department, Nursing School Coimbra, Coimbra, 3046-851, Portugal; ^2^Public Health Nursing Department, Federal University of Rio de Janeiro, Rio de Janeiro, RJ 21941-901, Brazil; ^3^Emergency Department, Central Hospital Dr. Ayres Meneses, São Tomé and Príncipe

**Correspondence:** Teresa Barroso - tbarroso@esenfc.pt

*Addiction Science & Clinical Practice* 2016, **11(Suppl 1)**: A52

**Background**: The harmful use of alcohol is a leading risk factor for morbidity, mortality and disability worldwide (WHO 2014). According to a local newspaper in São Tomé and Príncipe, 12–15 cases of accidents of different causes are admitted to the emergency department; however, their association with alcohol consumption is unknown. The aim of this study was to describe and analyze the sociodemographic profile and the types of alcohol-related accidents and episodes of violence among the patients who used the emergency department of the Central Hospital of São Tomé and Príncipe.

**Materials and methods**: Epidemiological, retrospective, exploratory, and descriptive study. The medical records of an emergency department of a reference hospital in São Tomé and Príncipe were analyzed, in a retrospective 13-month period. The patients whose reason for hospital admission was accident and/or violence were identified using a classification grid.

**Results**: Most of the victims were male, under the age of 30 years, single, and lived in Água Grande. Most incidents occurred on weekends, at night, and in public spaces. The main cause of injury was violence/assault, and most of the victims involved had intense alcoholic breath.

**Conclusions**: These results may contribute to the planning of local prevention programs targeted at individuals with the identified profile, as well as for to the development of alcohol-related public policies in Sao Tome and Principe.

## A53 **Substance use interventions in inpatient psychiatric settings**: **an opportunity missed?**

### Duncan Stewart

#### University of York, York, Yorkshire, YO10 5DD, UK

**Correspondence:** duncan.stewart@york.ac.uk

*Addiction Science & Clinical Practice* 2016, **11(Suppl 1)**: A53

**Background**: Misuse of alcohol and illicit drugs is a common problem among psychiatric patients. Admission to hospital represents an opportunity for staff to help patients with their substance use and related problems. Given a limited length of stay, screening and brief intervention (BI) may be a pragmatic option. A previous study reported a surprising lack of intervention by staff. This paper reports further analyses and discussion on the prospects of successfully delivering BIs in this challenging environment.

**Materials and methods**: Data were obtained from anonymised clinical records of patients admitted to four hospitals in London. All admissions during in 2012 were included. Incidents of substance use during admission were identified and details extracted. Staff actions which involved dialogue with the patient about their substance use were compared to other approaches.

**Results**: Talking to patients was associated with a lower rate of repeat incidents and was more likely for those involving alcohol than for other substances. There was evidence of clustering of staff actions by ward, but the type of intervention used was not predicted by patients’ personal or clinical characteristics.

**Conclusions**: The findings offer tentative evidence of the benefit of direct engagement with substance using patients admitted to psychiatric hospital. It seems feasible that brief interactions of this kind could form the basis for intervention development work and future evaluation.

## A54 Brief intervention for alcohol, tobacco and other drugs among users of primary care

### Angela Abreu^1^, Riany M. Brites^1^, Rafael Jomar^1^, Gerson Marinho^1^, Pedro Parreira^2^, Teresa Barroso^2^

#### ^1^Public Health Nursing, Federal University of Rio de Janeiro, Rio de Janeiro, RJ 21941-901, Brazil; ^2^Coimbra Nursing School, Coimbra, 3046-851, Portugal

**Correspondence:** Angela Abreu - angelabreu@globo.com

*Addiction Science & Clinical Practice* 2016, **11(Suppl 1)**: A54

**Background**: In general, drug users seek specialized services in a fairly advanced stage of consumption. Therefore, it is considered that the intervention at early stages problem improves the prognosis and, therefore, it is necessary to develop detection strategies and Brief interventions, such as screening the population susceptible to the use of these substances. Thus, care in Primary Health Care is a privileged opportunity for that screening and early monitoring.

**Objective:** To Screen the use of alcohol, tobacco and other drugs among users of primary care to identify factors associated with the need for intervention over these substances.

**Methods:** Cross-sectional study developed with 1489, primary health care users in Rio de Janeiro, Brazil, who responded to the Alcohol, Smoking and Substance Involvement Screening Test. A multivariate logistic regression was conducted to identify sociodemographic characteristics associated with the need for intervention by the use of alcohol, tobacco and other drugs.

**Results:** The high consumption by these substances by the respondents were described as alcohol, tobacco, and among street drugs, marijuana. In both genders, a greater chance of need for intervention by the use of tobacco and alcohol were found among people with low education, high incomes and who had no religion. Women were more likely to receive any intervention due to tobacco consumption.

**Conclusion**: These results showed socioeconomic characteristics as indicators of alcohol and tobacco intervention. Thus, primary care professionals should systematically screen the use of these substances for an early identification of people in need of Brief intervention.

## A55 Alcohol and drug risk patterns of patients screened by advanced practice registered nursing (APRN) students

### J. Paul Seale^1^, J. Aaron Johnson^2^, Dena Henry^2^, Sharon Chalmers^3^, Freida Payne^4^, Linda Tuck^5^, Akula Morris^1^

#### ^1^Family Medicine, Mercer University, Macon, GA 31206, USA; ^2^Institute of Public & Preventive Health, Augusta University, Augusta, GA 30912, USA; ^3^School of Nursing, University of North Georgia, Dahlonega, GA 30597, USA; ^4^College of Nursing, Mercer University, Atlanta, GA 30341, USA; ^5^Department of Nursing, Armstrong Atlantic State University, Savannah, GA 31419, USA

**Correspondence:** J. Paul Seale - seale.paul@navicenthealth.org

*Addiction Science & Clinical Practice* 2016, **11(Suppl 1)**: A55

**Background**: In clinical settings not offering universal alcohol and drug screening, APRN students receiving SBIRT training make choices regarding which patients to screen during clinical rotations. Little information is available on risk levels of patients screened by APRN students as they are learning how to perform screening, brief intervention and referral to treatment (SBIRT).

**Materials and methods**: Within a U.S. SBIRT training consortium, APRN students were required to practice SBIRT with multiple patients in clinical settings. Students completed assessment logs, documenting results of single item alcohol & drug screening questions; AUDIT or DAST responses, if indicated; and brief intervention steps completed.

**Results**: 113 students provided SBIRT services to 538 patients (mean age44; SD 15.0; 53.5 % female). Positive single question alcohol screens were more frequent than positive single question drug screens (55.3 vs. 25.5 %). Mean AUDIT score of patients with positive alcohol screens was 6.5(range 0–33). Mean DAST score of patients with positive drug screening responses was 1.5(range 0–10). Using previously validated AUDIT cut-points, 42.2 % of SASQ positive patients were low risk, 42.2 % were moderate risk, and 15.6 % were high risk. Existing DAST cut-points classified 57.4 % of drug screen-positive patients as low risk, 14.9 % as moderate risk, and 27.7 % as high risk.

**Conclusions**: Positive screening rates for patients screened by nursing students were higher than reported rates in most healthcare settings, providing ample opportunities for practicing SBIRT skills. Students encountered many patients with moderate alcohol risk who were likely to benefit from brief intervention. Numerous patients with high risk drug scores may have presented a greater challenge to novice students’ SBIRT skills, suggesting a need for clinical supervisors skilled in SBIRT. Overall, students selected patients across the spectrum of alcohol and drug use, providing excellent opportunities for learning SBIRT skills.

## A56 **Effects of alcohol screening and brief interventions among drug users in treatment**: **pre-experimental study**

### Teresa Barroso^1^, Cátia Gonçalves^2^

#### ^1^Mental Health Nursing Department, Nursing School of Coimbra, Coimbra, 3046-851, Portugal; ^2^ARS-Centro, Coimbra, 3020, Portugal

**Correspondence:** Teresa Barroso - tbarroso@esenfc.pt

*Addiction Science & Clinical Practice* 2016, **11(Suppl 1)**: A56

**Background**: There is a substantial body of evidence which shows that Screening and Brief Interventions (SBIs) in primary care settings can be effective. However, the dissemination and implementation of SBIs in other settings are less clear. Alcohol abuse by drug users after withdrawal treatment can compromise adherence to treatment. It is essential to guide efforts to develop, evaluate and treat alcohol consumption problems among addicts. Screening for alcohol problems should become a routine assessment in any context of nursing practice. Objective: To assess the effect of SBIs developed by the nurse specialist in mental health in reducing the risk of excessive alcohol consumption among drug users in treatment.

**Materials and methods**: A pre-experimental study was conducted with a single group, and assessment before and after the intervention (4-month follow-up). The sample consisted of 15 patients (14 men and 1 women) with a mean age of 33 years (SD = 9.2). The data collection tool was a structured interview using a questionnaire, including the AUDIT to assess the level of risk of alcohol consumption. The subjects’ informed consent was obtained. Follow-up occurred at 4 months after the intervention. Interventions were conducted according to a previously designed protocol based on the risk level identified. Data were analyzed using the Wilcoxon Signed-Rank Test.

**Results**: At baseline: 6 subjects were in risk zone I, 6 in risk zone II, and 3 in risk zone III. After the 4-month follow-up, 11 subjects were in risk zone I and 4 were in risk zone II. A positive statistically significant effect was found in the participants’ progress concerning the risk level (Z = −2.828, p = .0025).

**Conclusions**: SBIs reduced and stabilized the risk levels of alcohol consumption among drug users. These findings suggest the importance of implementing SBIs in other health care settings.

## A57 Problematic and pathological Internet use - Development of a short screening questionnaire

### Anja Bischof, Hans-Juergen Rumpf, Bettina Besser, Gallus Bischof

#### Department of Psychiatry and Psychotherapy, University of Luebeck, Luebeck, D-23538, Germany

**Correspondence:** Anja Bischof - anja.bischof@uksh.de

*Addiction Science & Clinical Practice* 2016, **11(Suppl 1)**: A57

**Background**: Problematic and pathological Internet use represents an increasing challenge in modern society. The addiction treatment system reaches only a small number of the affected individuals. Because treatment-seeking is marginal there is an increased requirement to improve case detection in pro-active settings. Existing screening instruments are often impractical to use and time-consuming as well as not validated on grounds of clinical criteria. The aim of this study was to develop an optimized short screening questionnaire for identifying problematic and pathological Internet use.

**Materials and methods**: Two samples (N = 3040; N = 1209) recruited via systematic pro-active screening in the settings of vocational schools and job centers have been used. Participants were screened with the Compulsive Internet Use Scale (CIUS). A fully standardized diagnostic interview served as gold standard for Internet-related disorders. Regression analyses were used to examine the performance of CIUS-Items in four random samples.

**Results**: Based on regression models, two different short versions of the CIUS with 5 and 7 items have been developed and were compared. Sensitivity and specificity are displayed in ROC-curves. The AUC-values of both short screenings are high (0.96, 0.97). χ^2^ tests according to McClish show that both short screenings do not differ significantly from each other and in comparison to the original test (p = .48, .52, .20). The shorter five item test version was chosen as new CIUS short screening.

**Conclusions**: The analysis showed that the performance of the CIUS short screening is just as valid in detecting clinically significant symptoms of problematic or pathological internet use as the performance of the original CIUS. These results can be used within the framework of SBI-approaches and further research.

## A58 Establishing a standard joint unit

### Cristina Casajuana^1,2^, Hugo López-Pelayo^1,3^, María Mercedes Balcells^1^, Lídia Teixidó^1^, Laia Miquel^1,2^, Joan Colom^4^, Antoni Gual^1,2^

#### ^1^Grup de Recerca en Adiccions Clínic (GRAC), Addictions Unit, Department of Psychiatry, Clinical Institute of Neuroscience, Hospital Clínic, Universitat de Barcelona, Red de Trastornos Adictivos (RTA), Barcelona, 08036, Spain; ^2^Institut d’Investigacions Biomèdiques August Pi i Sunyer (IDIBAPS), Barcelona, 08036, Spain; ^3^Fundació Clínic per la Recerca Biomèdica, Barcelona, 08036, Spain; ^4^Agència de Salut Pública de Catalunya, Departament de Drogodependències, Generalitat de Catalunya, Barcelona, 08002, Spain

**Correspondence:** Cristina Casajuana - casajuana@clinic.cat

*Addiction Science & Clinical Practice* 2016, **11(Suppl 1)**: A58

**Background**: Identification of cannabis users eligible for early intervention remains complex. Risky cannabis use keeps poorly defined and when assessing cannabis use, quantities rest often unexplored as no reliable registration systems exist. Standard units offer the possibility to overcome these limitations. We propose to establish a Standard Joint Unit (SJU) based on cannabis main constituents with implication on health—9-Tetrahydrocannabinol (THC) and Cannabidiol (CBD). Preliminary results based on 125 samples are presented.

**Materials and methods**: Naturalistic prospective study with current cannabis users of four subpopulations (universities, nightclubs, out-patient mental health service and cannabis associations) in Barcelona. We designed and administered a questionnaire on cannabis use patterns and asked participants to donate one joint for analysis (with approval of the Ethic Committee of the Hospital Clínic). Quantification of THC and CBD proceeded according to the recommendations of the UNODC using HPLC–UV. Statistical analyses were done with SPSS v20.

**Results**: Of 125 joints analyzed, 32 contained hashish and 93 marihuana. Median THC quantity was 40 % higher in hashish joints than in marihuana joints (7.0 mg THC (IQR 12.2 mg THC) versus 4.1 mg THC (IQR 8.5 mg THC)). Median CBD quantity was 3.4 mg (IQR 4.4 mg CBD) in hashish joints and 0.01 mg CBD (IQR 0.02 mg CBD) in marihuana joints.

**Conclusions**: To define cannabis risky use and facilitate early intervention, quantities consumed should no longer be ignored. A SJU accounts for the quantity of cannabis main constituents in its most used administration form, being of great value to advance in the study of which cannabis patterns are risky. Our preliminary results also show marked CBD differences between hashish and marihuana joints that need to be further explored.

## A59 Does documented brief intervention predict decreases in drinking among patients with risky drinking in routine primary care?

### Kimberly A. Hepner^1^, Katherine J. Hoggatt^2^, Andy Bogart^1^, Susan M. Paddock^1^

#### ^1^RAND Corporation, Santa Monica, CA, 90407, USA; ^2^VA HSR&D Center for the Study of Healthcare Innovation, Implementation and Policy, VA Greater Los Angeles Healthcare System, Sepulveda, CA, 91343, USA

**Correspondence:** Kimberly A. Hepner - hepner@rand.org

*Addiction Science & Clinical Practice* 2016, **11(Suppl 1)**: A59

**Background**: We evaluated whether brief intervention (BI), as documented in the medical record, predicts decreases in drinking 6 months after a positive screen for alcohol misuse.

**Materials and methods**: We enrolled patients who recently screened positive for alcohol misuse during a routine annual screen in primary care, and who had not received treatment for alcohol misuse within the past 3 months. We conducted medical record review to assess whether providers documented advice to reduce or abstain (advice), feedback about risks of alcohol use to health (risk feedback), feedback about how patient drinking compares to norms or recommended limits (normative feedback), or discussion of drinking related goals (goal discussion). Every instance of each documented element was coded from 7 days before the date of the positive alcohol screen to 60 days after. We conducted baseline and 6-month follow-up telephone interviews and computed the percentage of heavy drinking days in the past 30 days at each time point. We fit four separate regression models, one examining each BI element. We fit another model that included the total count of instances of any combination of elements.

**Results**: Of the 528 patients included, 87 % had at least one documented instance of receiving advice, 88 % had risk feedback, 59 % had normative feedback, 38 % had goal discussion, and 75 % had 3 or more instances of any combination of elements of BI. In the individual element models, none of the elements were significantly associated with a decrease in heavy drinking. The total number of instances of any BI elements did not predict decreased heavy drinking.

**Conclusions**: Results suggest that provider documentation of elements of BI and increasing numbers of instances of BI elements were not associated with decreased heavy drinking at 6-month follow-up among patients identified with risky drinking.

## A60 **Use of financial incentives to implement alcohol consumption recording in primary health care among adults with schizophrenia and other psychoses**: **a cross-sectional and retrospective cohort study**

### Zarnie Khadjesari^1,2^, Sarah L. Hardoon^1^, Irene Petersen^1^, Fiona L. Hamilton^1^, Irwin Nazareth^1^

#### ^1^Department of Primary Care and Population Health, University College London, London, WC1E 6BT, UK; ^2^Addictions Department, King’s College London, London, WC2R 2LS, UK

^1^Department of Primary Care and Population Health, University College London, London, WC1E 6BT, UK; ^2^Addictions Department, King’s College London, London, WC2R 2LS, UK**orrespondence:** zarnie.khadjesari@kcl.ac.uk

*Addiction Science & Clinical Practice* 2016, **11(Suppl 1)**: A60

**Background**: Lack of financial incentive is a frequently cited barrier to alcohol screening in primary care. The Quality and Outcomes Framework (QOF) payment for performance scheme has reimbursed UK primary care practices for alcohol screening in people with schizophrenia since April 2011. This study aimed to determine the impact of financial incentives on alcohol screening by comparing rates of alcohol recording in people with and without schizophrenia between 2000 and 2013.

**Method**: Cross-sectional and retrospective cohort study. Alcohol data were extracted from The Health Improvement Network (THIN) database of UK primary care records using Read Codes for level of alcohol consumption, continuous measures of drinking (e.g. units a week), and Read Codes for types of screening test.

**Results**: A total of 14,860 individuals (54 % (8068) men and 46 % (6792) women) from 409 general practices aged 18–99 years with schizophrenia were identified during April 2011 to March 2013. Of these, 11,585 (78 %) had an alcohol record, of which 99 % (8150/8257) of Read codes for level of consumption were eligible for recompense in the QOF. There was a 839 % (more than eightfold) increase in alcohol recording among people with schizophrenia over the 13 year period [rate ratio per annum increase 1.19 (95 % CI 1.18–1.20)], compared with a 62 % increase among people without a serious mental illness [rate ratio per annum increase 1.04 (95 % CI 1.03–1.05)].

**Conclusion**: Financial incentives offered by the serious mental illness QOF appear to have a substantial impact on alcohol screening in UK primary care.

## A61 Validation of an internet-based AUDIT-C in adults seeking help with their drinking online

### Zarnie Khadjesari^1,2^, Ian R. White^3^, Jim McCambridge^4^, Louise Marston^1^, Paul Wallace^1^, Christine Godfrey^4^, Elizabeth Murray^1^

#### ^1^Department of Primary Care and Population Health, University College London, London, WC1E 6BT, UK; ^2^Addictions Department, King’s College London, London, WC2R 2LS, UK; ^3^MRC Biostatistics Unit, Cambridge, Cambridgeshire, CB2 0SR, UK; ^4^Department of Health Sciences, University of York, York, Yorkshire, YO10 5DD, UK

**Correspondence:** zarnie.khadjesari@kcl.ac.uk

*Addiction Science & Clinical Practice* 2016, **11(Suppl 1)**: A61

**Background**: The abbreviated Alcohol Use Disorder Identification Test for Consumption (AUDIT-C) is rapidly becoming the alcohol screening tool of choice for busy practitioners in clinical settings and by researchers keen to limit assessment burden and reactivity. Cut-off scores for detecting drinking above recommended limits vary by population, setting, country and potentially format. This validation study aimed to determine AUDIT-C thresholds that indicated risky drinking among a population of people seeking help over the Internet.

**Method**: The data in this study were collected in the pilot phase of the Down Your Drink trial, which recruited people seeking help over the Internet and randomized them to a web-based intervention or an information-only website. Sensitivity, specificity, and positive and negative likelihood ratios were calculated for AUDIT-C scores, relative to weekly consumption that indicated drinking above limits and higher risk drinking. Receiver-operating characteristic (ROC) curves were created to assess the performance of different cut-off scores on the AUDIT-C for men and women. Past week alcohol consumption was used as the reference-standard and was collected via the TOT-AL, a validated online measure of past week drinking.

**Results**: AUDIT-C scores were obtained from 3720 adults (2053 female and 1667 male) searching the internet for help with drinking, mostly from the UK. The area under the ROC curve for risky drinking was 0.84 (95 % CI 0.80, 0.87) (Female) and 0.80 (95 % CI 0.76, 0.84) (Male). AUDIT-C cut-off scores for detecting risky drinking that maximize the sum of sensitivity and specificity were ≥8 for women and ≥8 for men; whereas those identifying the highest proportion of correctly classified individuals were ≥4 for women and ≥5 for men. AUDIT-C cut-off scores for detecting higher risk drinking were also calculated.

**Conclusions**: AUDIT-C cut-off scores for identifying alcohol consumption above weekly limits in this largely UK based study population were substantially higher than those reported in other validation studies. Researchers and practitioners should select AUDIT-C cut-off scores according to the purpose of identifying risky drinkers and hence the relative importance of sensitivity and/or specificity.

## A62 Could the computer-assisted brief intervention be an alternative to face-to-face approach?

### Hana Sovinová^1^, Ladislav Csémy^1,2^

#### ^1^Center for Coordination, Monitoring and Research on Alcohol and Tobacco, National Institute of Public Health, Prague, Bohemia, 100 42, Czech Republic; ^2^Center for Epidemiological and Clinical Research of Addictions, National Institute of Mental Health, Klecany, Bohemia, 250 67, Czech Republic

**Correspondence:** Hana Sovinová - hana.sovinova@szu.cz

*Addiction Science & Clinical Practice* 2016, **11(Suppl 1)**: A62

**Background**: Computer-Assisted Brief Interventions (CABI) are online alcohol interventions useful for groups less likely to access traditional alcohol-related advice. The goal of the study was to explore the usability of online alcohol intervention when referred by patient’s GP.

**Materials and methods**: The study was part of the EU BISTAIRS project. AUDIT-C was used as screening tool for alcohol problems. 15 GPs from nine localities participated in the study. All were appropriately trained in methods of screening and brief intervention. German web-based application TrinkCheck was adapted to Czech as CABI. Doctors were instructed to refer half of those screened positive to online intervention.

**Results**: In sum 531 patients were asked to participate in the study. 312 (58.7 %) agreed and completed the screening. 142 (45.5 %) patients (84 males, 58 females) were found positive on AUDIT-C screening test (≥5 for males, and ≥4 for females). 83 (58.4 %) of these were referred to online alcohol intervention TrinkCheck; 56 (39.4 %) patients received brief intervention face-to-face; 3 declined the brief advice. Online application was completed by 56 patients (67.5 % out of 83 assigned to the online intervention); 64.2 % reported it as “useful” or “very useful”.

**Conclusions**: Findings of this pilot study suggest acceptable adherence of patients to doctor’s advice to complete the online alcohol intervention. Precondition for application of CABI is a good availability of internet. Limitation of this study is that effects of two intervention modes were not compared. In international literature few studies report on impact of online alcohol interventions and the findings do not allow concluding that CABI is as effective as traditionally delivered SBI. Online alcohol intervention may serve as supplement supporting face-to–face intervention, for example when face-to-face intervention is not practical.

